# Delirium after surgery: a retrospective study of predictors, complications, and screening patterns in the national surgical quality improvement program

**DOI:** 10.1016/j.eclinm.2025.103629

**Published:** 2025-11-07

**Authors:** Adriana C. Panayi, Sarah Friedrich, Jasmin Rühl, Thomas Schaschinger, Tobias Niederegger, Leonard Knoedler, Samuel Knoedler, Carsten Rendenbach, Sascha Treskatsch, Leila Harhaus, Max Heiland, Dennis P. Orgill, Gabriel Hundeshagen

**Affiliations:** a Charité – Universitätsmedizin Berlin, Corporate Member of Freie Universität Berlin, Humboldt-Universität zu Berlin, and Berlin Institute of Health, Department of Oral and Maxillofacial Surgery, Berlin, Germany; b Department of Hand, Replantation, and Microsurgery BG Klinikum Unfallkrankenhaus Berlin and Chair of Hand, Replantation, and Microsurgery at the Charité Universitätsmedizin Berlin, Germany; c Department of Mathematical Statistics and Artificial Intelligence in Medicine, University of Augsburg, Augsburg, Germany; d Medical Faculty, University of Heidelberg, Heidelberg, Germany; e Division of Plastic Surgery, Department of Surgery, Brigham and Women's Hospital, Harvard Medical School, Boston, MA, USA; f Charité – Universitätsmedizin Berlin, Corporate Member of Freie Universität Berlin and Humboldt-Universität zu Berlin, Department of Anaesthesiology and Intensive Care Medicine, Campus Benjamin Franklin, Hindenburgdamm 30, 12203, Berlin, Germany; g Department of Hand, Plastic and Reconstructive Surgery, Burn Center, BG Trauma Center Ludwigshafen, University of Heidelberg, Ludwigshafen, 67071, Germany

**Keywords:** Quality improvement, Surgery, Delirium, Prognostic, Risk factor, Critical care

## Abstract

**Background:**

Postoperative delirium is a serious yet underrecognized complication affecting diverse surgical populations, with profound implications for morbidity, mortality, and long-term cognitive function. Its prediction remains imprecise, and screening practices vary widely.

**Methods:**

We conducted a retrospective analysis of the 2021–2023 data from the American College of Surgeons National Surgical Quality Improvement Program (ACS-NSQIP). The study included 217,783 adult surgical patients with documented delirium assessment. Patients were categorized as delirium, non-delirium, or unscreened, with an additional 2.7 million unscreened patients analyzed to assess selection bias in screening. The primary outcome was the incidence of delirium. Other outcomes included surgical and medical complications, mortality, length of stay, functional decline, discharge destination and conditions, and perioperative lab values. Multivariable regression models were used to identify independent predictors of delirium and its associated outcomes.

**Findings:**

Delirium screening was performed exclusively in patients aged ≥75 years, accounting for 7.3% of all surgical patients. Screening rates declined with advancing age (44% of patients aged 90+ vs 56% aged 75–79), while the proportion screening positive increased sharply (3.1% at 75–79 years vs 12.8% at ≥90 years). Delirium occurred in 10.6% (n = 23,100) of screened patients. Compared with non-delirium patients, those with delirium were older (mean 81.3 vs 79.9 years, p < 0.0001), more functionally dependent, and had higher ASA class and comorbidity burden. Dementia (37% vs 7.9%, p < 0.0001), recent falls (40% vs 18%, p < 0.0001), and urgent/emergency surgery (55% vs 26%, p < 0.0001) were strongly associated. Each 10-min increase in operative time was seen to independently be associated with 2.3% raised odds of delirium (p < 0.0001). Delirium was independently associated with higher 30-day mortality (OR 3.2, 95% CI 2.9–3.5), reoperation (OR 2.3, 95% CI 2.1–2.5), surgical complications (OR 1.8, 95% CI 1.6–1.9), loss of independence (OR 1.6, 95% CI 1.5–1.7), and reduced odds of home discharge (OR 0.5, 95% CI 0.4–0.5).

**Interpretation:**

Postoperative delirium is an independent predictor of adverse surgical outcomes yet remains substantially under-screened, with disparities across patient groups. These findings underscore the need for standardized, routine screening and targeted prevention strategies to improve perioperative care.

**Funding:**

No funding was received for this study.

**Research in context**

**Evidence before this study**

Postoperative delirium is widely recognized as a common complication in older surgical patients, associated with increased mortality, morbidity, prolonged hospital stay, functional decline, and institutionalization. However, existing studies are often limited to single centers, select surgical subspecialties, or geriatric cohorts, restricting generalizability. Previous work has emphasized the utility of screening tools, yet screening practices remain highly variable across institutions. Large-scale epidemiologic data capturing delirium incidence, predictors, outcomes, and disparities in screening across the full spectrum of surgical populations are scarce. Moreover, little is known about how selection bias in delirium screening may influence our understanding of its true burden in surgical practice.

**Added value of this study**

Using data from the American College of Surgeons National Surgical Quality Improvement Program (ACS-NSQIP), this is the first multicenter analysis of more than 217,000 screened surgical patients and over 2.7 million unscreened patients to evaluate postoperative delirium across diverse surgical settings. We identify independent perioperative predictors of delirium, quantify its strong association with adverse outcomes—including a more than three-fold increase in 30-day mortality—and demonstrate substantial disparities in who receives screening. Importantly, we show that only 7.3% of surgical patients were formally assessed for delirium, with clear selection bias favoring older, frailer patients. Our findings highlight the magnitude of under-screening, the limitations of current practices, and the clinical consequences of missed diagnoses.

**Implications of all the available evidence**

This study demonstrates that postoperative delirium is both common and highly consequential but remains under-recognized in surgical care, with systematic disparities in screening. By establishing delirium as an independent predictor of mortality, morbidity, and functional decline across a large, heterogeneous surgical cohort, our results underscore the urgent need for routine, standardized screening and targeted prevention strategies. Integrating delirium surveillance into perioperative quality programs could help reduce preventable harm, improve functional recovery, and support equitable perioperative care.

## Introduction

Postoperative delirium is an often-underestimated condition that can dramatically alter the course of surgical recovery.^1^ A survey by the American Geriatrics Society's Geriatrics-for-Specialists Initiative (AGS-GSI) found that surgical specialists ranked delirium as the most “essential” issue in the care of older adults, while also identifying it as the least understood geriatric condition with the largest knowledge gap in optimal management.^2^

Characterized by acute fluctuations in attention, consciousness, and cognition, delirium typically arises within hours to days after an operation and can affect patients of all ages—though it is particularly prevalent in older adults and those with pre-existing cognitive impairment.^3^^,^^4^ Its clinical presentation is heterogeneous, ranging from hyperactive agitation to hypoactive withdrawal, making recognition challenging. Delirium can be identified through standardized instruments. Screening tools such as the Four As Test (4AT) and the Nursing Delirium Screening Scale (Nu-DESC) are designed to identify patients at risk and should be followed by formal diagnostic assessment, whereas the Confusion Assessment Method (CAM) is widely used as a diagnostic tool that evaluates key features including acute onset, inattention, disorganized thinking, and altered level of consciousness. While its presentation may appear transient, the long-term repercussion are far-reaching: an increased morbidity and mortality, prolonged hospitalization, greater healthcare burden, and a higher risk of long-term cognitive decline including dementia have been proposed.^5^, ^6^, ^7^, ^8^, ^9^, ^10^, ^11^, ^12^, ^13^

Despite growing awareness of its clinical significance—for example, through initiatives like Delirium Awareness Day—the underlying mechanisms and risk factors for delirium remain incompletely understood, and its prediction imprecise.^14^ The literature remains fragmented, with a vast and often inconsistent array of proposed prognostic and care-related risk factors—highlighting the lack of consensus on which variables hold the greatest clinical significance.^15^ With the exception of a few recent meta-analyses,^16^, ^17^, ^18^, ^19^, ^20^ few of the proposed risk factors have been consistently validated in large, diverse cohorts.^8^ Therefore, much of the current evidence stems from studies with limited sample sizes, single-center designs, and a narrow focus on specific surgical specialties or patient populations.

This study aims to leverage data from the American College of Surgeons National Surgical Quality Improvement Program (ACS-NSQIP) to address these gaps by conducting the first large-scale, multicentric investigation of postoperative delirium across a heterogeneous surgical population. This work represents a critical step toward a more unified understanding of postoperative delirium, transcending the boundaries of individual surgical disciplines and informing the development of more accurate and generalizable prognostic tools.

## Methods

### Data source

This study utilized data from the ACS-NSQIP, a nationally validated database that collects detailed perioperative data on surgical patients across participating institutions. The database is a prospective multicentric clinical registry. Participation in the NSQIP is voluntary, and the database includes more than 700 participating hospitals, the majority of which are large academic medical centers and tertiary referral institutions. Trained surgical clinical reviewers extract data from medical records using standardized definitions. The process undergoes routine inter-rater reliability audits to ensure accuracy. While the NSQIP is not population-based and therefore does not capture all surgical procedures performed nationally, it is one of the most comprehensive and rigorously validated surgical registries available. Importantly, the database is not publicly accessible; hospitals and researchers obtain access through institutional participation and formal data use agreements with the American College of Surgeons. Completeness and coverage vary depending on institutional resources and sampling strategies, but NSQIP has consistently demonstrated high data quality and reproducibility, making it widely used for outcomes research and benchmarking across surgical specialties.

### Ethics

The ACS-NSQIP and participating hospitals are responsible for patient consent and data collection. All patient information is de-identified prior to release, and the use of these data is therefore exempt from institutional review board (IRB) approval under the U.S. Department of Health and Human Services regulation 45 CFR 46.101(b) (4). Informed consent was obtained by participating institutions at the time of data collection, and no additional consent was required for this secondary analysis of de-identified data. Ethical approval for this analysis was obtained from Brigham and Women's Hospital, Boston, USA (IRB protocol number: 2013P001244).

### Patient selection

All adult patients (aged ≥18 years) recorded in the NSQIP Participant Use Data Files from the years 2021–2023 were screened. At the time of analysis these were the only years that included an assessment of postoperative delirium. Patients were included if they underwent surgery and had complete data for the “Delirium” variable. Patients were grouped into three categories:

Delirium Group: Patients with documented post-operative delirium.

Non-delirium Group: Patients screened for delirium but without a positive diagnosis.

Not Screened Group: Patients without documented delirium screening.

Delirium in NSQIP is defined as any documented episode occurring within 30 days postoperatively. The database does not provide information on the specific timing, screening tool used, or duration of delirium. The final analytic cohort consisted of the Delirium and Non-delirium group only, ensuring that all included patients were formally assessed for delirium. The Not Screened Group (unscreened patients) was analyzed separately in a supplementary analysis to evaluate potential selection bias, recognizing that patients not screened may systematically differ in risk profile and outcome from those included in the primary analysis.

### Variable extraction

A comprehensive list of extracted variables is presented in Supplemental Table S1. Creatinine change analyses were limited to 2022 and 2023 due to the timing of variable availability in the NSQIP dataset.

### Statistical analysis

All data were exported from the NSQIP datasets and managed using Microsoft Excel (V.16, Microsoft Corporation, USA) and SPSS (V.29; IBM Corporation, USA). Final statistical analyses were performed using Rv (V.4.4.1, R Foundation for Statistical Computing, Austria).

Continuous variables were compared using independent t-tests or Wilcoxon rank-sum tests as appropriate, and categorical variables using chi-square tests. For outcome modeling, multivariate linear regression was used to assess associations between delirium and continuous outcomes (operative time and length of stay), while logistic regression was employed for binary outcomes (e.g., mortality, complications, reoperation, readmission, infections, loss of independence, discharge disposition). Independent predictors in the multivariate models included delirium status, age, BMI, gender, obesity, COPD, CHF, bleeding disorder, preoperative transfusion, preoperative sepsis, dementia, fall within the last six months, American Society of Anesthesiologists Physical Status Classification System (ASA) class, origin location (home/permanent residence, acute area hospital, other facility, unknown) urgency of surgery (elective or urgent/emergency), surgical setting (inpatient/outpatient), and year of surgery. A check for multicollinearity among predictors was performed using the variance inflation factor (VIF) method. All p values and 95% confidence intervals (CIs) for the primary predictor—delirium status—were adjusted for multiple comparisons using Bonferroni correction. Subgroup analyses included comparisons between the primary cohort and the excluded unscreened group to investigate potential selection bias in delirium screening across institutions, demographic groups, and surgical specialties.

### Role of funding source

No funding was received for this study.

## Results

### Variables associated with delirium assessment

**Fig. 1:**
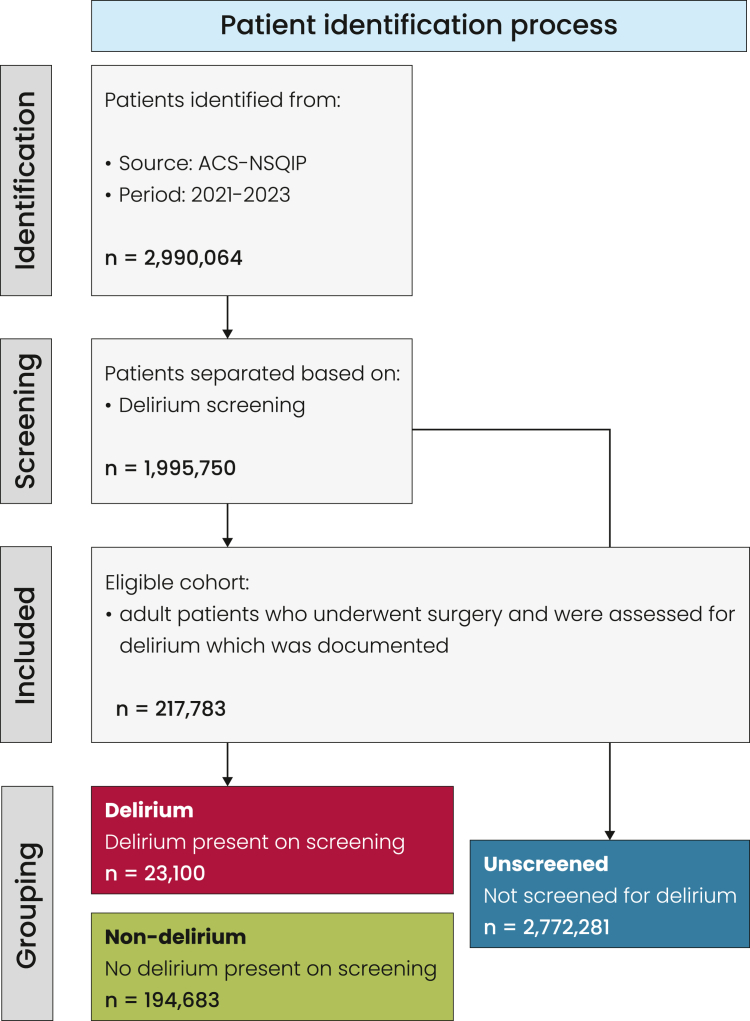
**Patient selection process and grouping**.

**Fig. 2:**
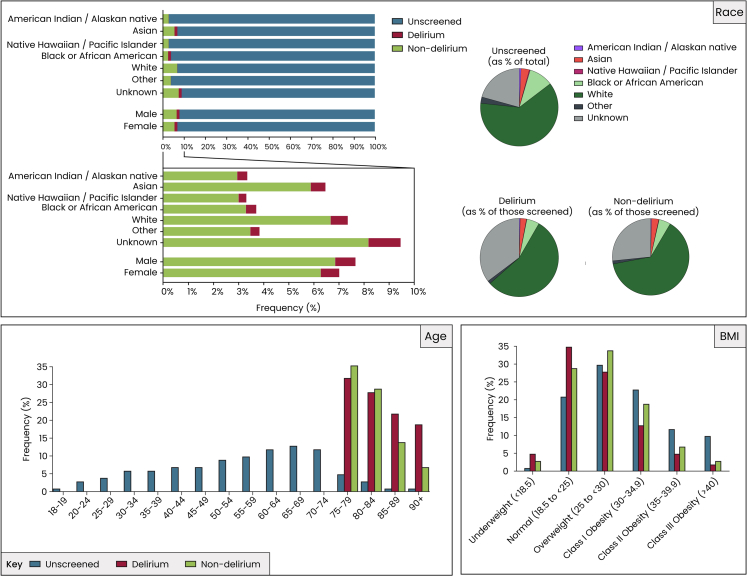
**Age, BMI, gender and race distribution of the three groups.** The data are categorized into three groups: unscreened, screened with delirium, and screened without delirium. The top left panel bar charts show the proportion of unscreened, delirium, and non-delirium patients by race and sex. Most patients were White, followed by Black or African American, with most unscreened being female. The pie charts show the distribution of race among unscreened patients, among those diagnosed with delirium and among those screened but without delirium. The bottom left panel is an age frequency histogram showing screening per age group. In the two oldest age groups, screening was more likely to result in a positive diagnosis. The bottom right panel is a BMI distribution histogram showing that screening for delirium was highest in the overweight category, while screening for delirium was more likely to result in a positive diagnosis in the underweight and normal weight range.

Of a total of 2,990,064 patients included in the NSQIP, 7.3% (n = 217,783) underwent an assessment for delirium (Supplemental Tables S2–S5; Figs. 1 and 2). No patients younger than 75 years old were screened, meaning that on average the patients that underwent an assessment for delirium were significantly older (mean 80 vs 55 years), with a much higher proportion aged >90. Of the total 2,990,064 patients, delirium screening was almost exclusively performed in those aged 75 years and older. In patients younger than 75, virtually all were unscreened (>99.9%). Among those aged 75–79 years (n = 236,001), 56.3% were unscreened, 3.1% screened positive for delirium, and 40.6% screened negative. For patients aged 80–84 years (n = 135,661), 53.8% were unscreened, 4.7% had delirium, and 41.5% screened negative. In those aged 85–89 years (n = 67,211), 50.6% were unscreened, 7.5% screened positive, and 41.9% screened negative. In the oldest group, patients aged ≥90 years (n = 33,499), 44.0% were unscreened, 12.8% screened positive, and 43.2% screened negative. These findings confirm that delirium screening was restricted to older patients, with screening rates declining modestly with increasing age, while the prevalence of delirium rose sharply across age strata (from 3.1% at 75–79 years to 12.8% at ≥90 years; Supplemental Table S6). BMI was lower in the assessment group (27.6 vs 30.5). There were significantly fewer Black (5% vs 10.1%) and Hispanic patients (12.9% vs 5.3%) in the assessment group, and women were less likely to be assessed (55.7% vs 57.9%). The assessment group had significantly higher rates of all co-morbidities assessed except for obesity and smoking. Patients from acute care hospitals or other facilities, and those who were partially or totally dependent prior to surgery were more likely to be assessed for delirium. In terms of surgical characteristics, there were more urgent/emergency cases in the assessment group (29% vs 17%), as well as more inpatient procedures (78% vs 46%). Screening rates increased steadily over the study period. In 2021, 65,822 of 983,851 patients (6.7%) underwent delirium screening, compared with 72,373 of 1,011,899 patients (7.2%) in 2022 and 79,588 of 994,314 patients (8.0%) in 2023 (Supplemental Table S3). This represents a relative increase of 7% from 2021 to 2022 and 11% from 2022 to 2023 (overall 19% increase from 2021 to 2023). Given the large sample sizes, these differences were statistically significant (p < 0.001), although overall screening rates remained low.

Patients who were assessed for delirium waited longer from admission to surgery, and had an over twice as long hospital stay (5.4 vs 2.4 days). They also had higher rates of reoperation, readmission, surgical and medical complications, and higher rates of partial or total dependency post-op (40% vs 25%).

### Comparison of screened patients with and without delirium

**Fig. 3:**
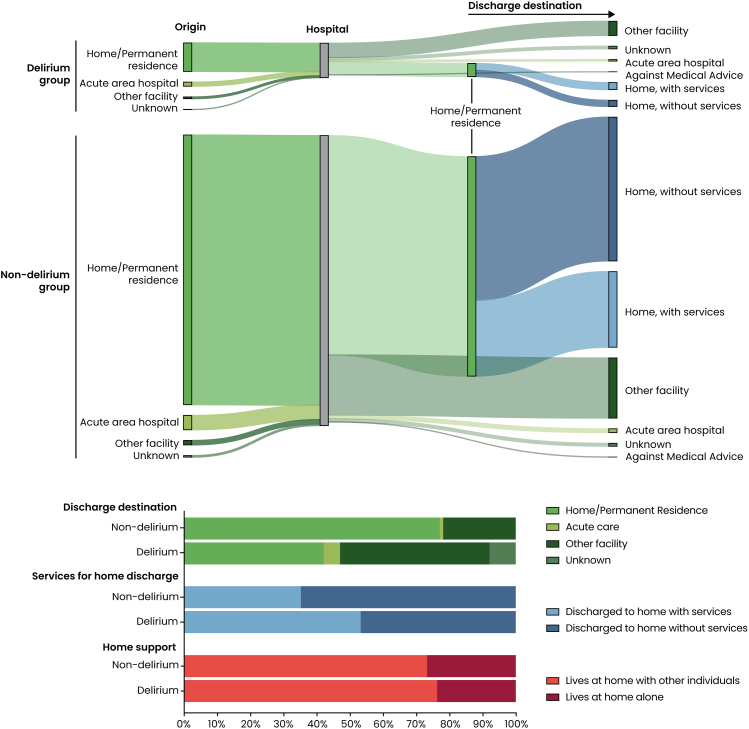
**Discharge destination.** The top panel is a Sankey visualization of the origin and destination of patients, with details on services required in case of home discharge. The bottom panel shows the distribution of patients in the different discharge destinations, whether they received services during home discharge and whether they had home support.

Of the total of 217,783 patients assessed for delirium, 23,100 (10.6%) developed the condition (Fig. 1; Supplemental Table S7). Patients with delirium were significantly older than those without (81.3 ± 4.2 vs 79.9 ± 3.9 years, p < 0.0001), with a higher proportion aged over 90 years (18.5% vs 7.4%, p < 0.0001; Table 1; Fig. 2). Focusing on pre-operative health and co-morbidities, patients who developed delirium were significantly more likely to have a higher ASA class, with more patients having life-threatening and moribund status (p < 0.0001). They were significantly more likely (p < 0.0001) to have diabetes, insulin-treated diabetes, COPD, ascites, be dialysis-dependent patients, need preoperative transfusion, have preoperative sepsis, be ventilator dependent prior to surgery, be current smokers, and have disseminated cancer. The two co-morbidities found to differ the most between the two groups were dementia (37.0% vs 7.9%; p < 0.0001), and a history of a fall within the last six months (40.2% vs 18.4%; p < 0.0001). Obesity was less prevalent among patients who developed delirium (23.1% vs 30.3%; p < 0.0001), while hypertension was similarly prevalent in both groups (71.8% vs 72.1%; p = 1.00). The origin of the patients also differed, with more patients with delirium being admitted from acute area hospitals or other facilities (16.9% vs 6.5%; p < 0.0001; Fig. 3). Delirium patients were also more likely to be partially or totally dependent before surgery (p < 0.0001).

**Table 1:** Baseline factors.

Demographics and characteristics	Overall (n = 217,783)	Delirium (n = 23,100)	Non-delirium (n = 194,683)	Adjusted p value
Age, mean ± SD	80.0 ± 4.0	81.3 ± 4.2	79.9 ± 3.9	**<0.0001**
Age >90	18,766 (8.6)	4280 (18.5)	14,486 (7.4)	**<0.0001**
BMI (kg/m^2^), mean ± SD	27.6 ± 5.8	26.4 ± 5.9	27.8 ± 5.7	**<0.0001**
**Gender**				1.00
Female	121,201 (55.7)	12,865 (55.7)	108,336 (55.7)	
Male	96,576 (44.4)	10,233 (44.3)	86,343 (44.4)	
**Race**				**<0.0001**
American Indian or Alaskan native	689 (0.3)	83 (0.4)	606 (0.3)	
Asian	6606 (3.0)	604 (2.6)	6002 (3.1)	
Native Hawaiian or Pacific Islander	338 (0.2)	32 (0.1)	306 (0.2)	
Black or African American	10,770 (5.0)	1204 (5.2)	9566 (4.9)	
White	136,489 (62.7)	12,743 (55.2)	123,746 (63.6)	
Other (Some other race/Race combinations with low frequency)	2893 (1.3)	279 (1.2)	2614 (1.3)	
Unknown	59,998 (27.6)	8155 (35.3)	51,843 (26.6)	
**Ethnicity: Hispanic**	8401 (5.3)	861 (5.6)	7540 (5.2)	1.00
**ASA class**				**<0.0001**
1—No disturbance	670 (0.3)	26 (0.1)	644 (0.3)	
2—Mild disturbance	42,377 (19.5)	1923 (8.4)	40,454 (20.8)	
3—Severe disturbance	137,507 (63.3)	13,757 (59.8)	123,750 (63.7)	
4—Life-threatening	35,585 (16.4)	6925 (30.1)	28,660 (14.8)	
5—Moribund	1094 (0.5)	379 (1.7)	715 (0.4)	
**Preoperative health and comorbidities**				
Obesity	60,708 (29.6)	4679 (23.1)	56,029 (30.3)	**<0.0001**
Diabetes	45,921 (21.1)	5159 (22.3)	40,762 (20.9)	**0.0001**
Insulin treated	14,175 (30.9)	1898 (36.8)	12,277 (30.1)	**<0.0001**
COPD	17,623 (8.1)	2542 (11.0)	15,081 (7.8)	**<0.0001**
CHF	20,673 (9.5)	3329 (14.4)	17,344 (8.9)	**<0.0001**
Ascites	1956 (0.9)	438 (1.9)	1518 (0.8)	**<0.0001**
Dialysis	2407 (1.1)	449 (1.9)	1958 (1.0)	**<0.0001**
Bleeding disorder	19,023 (8.7)	3167 (13.7)	15,856 (8.1)	**<0.0001**
Transfusion	4282 (2.0)	1001 (4.3)	3281 (1.7)	**<0.0001**
Pre-operative sepsis	19,957 (9.2)	4457 (19.3)	15,500 (8.0)	**<0.0001**
Ventilator dependent	762 (0.4)	322 (1.4)	440 (0.2)	**<0.0001**
Hypertension	156,917 (72.1)	16,591 (71.8)	140,326 (72.1)	1.00
Current smoker	12,603 (5.8)	1728 (7.5)	10,875 (5.6)	**<0.0001**
Disseminated cancer	7108 (3.3)	915 (4.0)	6193 (3.2)	**<0.0001**
Corticosteroid use	11,587 (5.3)	1319 (5.7)	10,268 (5.3)	0.45
Dementia	23,842 (11.0)	8553 (37.0)	15,289 (7.9)	**<0.0001**
Fall within last six months	41,821 (20.7)	8621 (40.2)	33,200 (18.4)	**<0.0001**
Immunosuppressive Therapy				
Corticosteroids	6701 (3.1)	910 (3.9)	5791 (3.0)	**<0.0001**
Anti-rejection/transplant immunosuppressants	962 (0.4)	128 (0.6)	834 (0.4)	0.62
Synthetic DMARDs/DMDs	3326 (1.5)	253 (1.1)	3073 (1.6)	**<0.0001**
Biologic DMARDs/DMDs	1401 (0.6)	119 (0.5)	1282 (0.7)	0.93
Other	1019 (0.5)	114 (0.5)	905 (0.5)	1.00
**Origin Location**				**<0.0001**
Home/Permanent residence	200,185 (91.9)	19,118 (82.8)	181,067 (93.0)	
Acute area hospital	12,363 (5.7)	2731 (11.8)	9632 (5.0)	
Other facility	4142 (1.9)	1184 (5.1)	2958 (1.5)	
Unknown	1093 (0.5)	67 (0.3)	1026 (0.5)	
**Preoperative Functional Status**				**<0.0001**
Independent	192,994 (89.7)	16,474 (72.5)	176,520 (91.8)	
Partially Dependent	19,394 (9.0)	5159 (22.7)	14,235 (7.4)	
Totally Dependent	2679 (1.3)	1098 (4.8)	1581 (0.8)	
**Home support**				**<0.0001**
Lives at home with other individuals	140,076 (73.6)	14,089 (76.3)	125,987 (73.3)	
Lives alone at home	50,275 (26.4)	4373 (23.7)	45,902 (26.7)	
**Surgical urgency**				**<0.0001**
Elective	155,088 (71.2)	10,468 (45.3)	144,620 (74.3)	
Urgent/Emergency	62,695 (28.8)	12,632 (54.7)	50,063 (25.7)	
**Surgical specialty**				**0.04**
Cardiac	1480 (0.7)	266 (1.2)	1214 (0.6)	
General Surgery	74,140 (34.0)	7703 (33.4)	66,437 (34.1)	
Gynecology	5843 (2.7)	287 (1.2)	5556 (2.9)	
Neurosurgery	12,152 (5.6)	1182 (5.1)	10,970 (5.6)	
Orthopedics	84,444 (38.8)	10,685 (46.3)	73,759 (37.9)	
Otolaryngology	2794 (1.3)	171 (0.7)	2623 (1.4)	
Plastics	896 (0.4)	62 (0.3)	834 (0.4)	
Thoracic	4653 (2.1)	301 (1.3)	4352 (2.2)	
Urology	15,645 (7.2)	898 (3.9)	14,747 (7.6)	
Vascular	15,638 (7.2)	1537 (6.7)	14,101 (7.2)	
Obstetrics	68 (0.0)	2 (0.0)	66 (0.0)	
Interventional Radiology	30 (0.0)	6 (0.0)	24 (0.0)	
**Type of Anesthesia**				**0.04**
General	171,637 (78.8)	18,810 (81.4)	152,827 (78.5)	
Monitored anesthesia care	17,257 (7.9)	1529 (6.6)	15,728 (8.2)	
Epidural	367 (0.2)	30 (0.1)	337 (0.2)	
Local	229 (0.1)	19 (0.21)	210 (0.1)	
Regional	2446 (1.1)	185 (0.8)	2261 (1.2)	
Spinal	25,686 (11.8)	2510 (10.9)	23,176 (11.9)	
None	15 (0.0)	4 (0.0)	11 (0.0)	
Other	140 (0.1)	13 (0.1)	127 (0.1)	
**Operative setting**				**<0.0001**
Inpatient	169,599 (77.9)	21,577 (93.4)	148,022 (76.0)	
Outpatient	48,184 (22.1)	1523 (6.6)	46,661 (24.4)	
**Year**				**<0.0001**
2021	65,822 (30.2)	8487 (36.7)	57,335 (29.5)	
2022	72,373 (33.2)	7676 (33.2)	64,697 (33.2)	
2023	79,588 (36.5)	6937 (30.0)	72,651 (37.3)	
**Time from admission to operation (days), mean** ± **SD**	0.9 ± 3.0	1.8 ± 4.6	0.8 ± 2.7	**<0.0001**
**Operative time (in minutes), mean** ± **SD**	122.5 ± 94.1	124.9 ± 106.1	122.2 ± 92.5	**0.02**
Urgent/Emergency	62,695 (28.8)	12,632 (54.7)	50,063 (25.7)	

SD, standard deviation; BMI, Body mass index; COPD, chronic obstructive pulmonary disease; CHF, congestive heart failure; DMARDs/DMDs, Disease-Modifying Antirheumatic Drugs/Disease-Modifying Drugs; ASA, American Society of Anesthesiologists; IQR, Interquartile range; n, number.

Reported as n (%), unless otherwise stated. Significant p values shown in bold. p values adjusted using Bonferroni correction.

Patients who developed post-operative delirium were significantly more likely to have undergone urgent or emergency procedures (54.7%) compared to those in the non-delirium group (25.7%; p < 0.0001; Table 2). Of the 12 surgical specialties assessed, two had a higher proportion in the delirium group (Orthopedics: 46.3% vs 37.9%; cardiac surgery: 1.2% vs 0.6%). General anesthesia was more frequently used in the delirium (81.4%) than in the non-delirium group (78.5%; p = 0.04; Table 2). In terms of care setting, delirium predominantly occurred in inpatient procedures (93.4% vs 76.0%). The proportion of delirium cases declined over time, from 36.7% in 2021 to 30.0% in 2023.

**Table 2:** Postoperative outcomes.

Outcomes	Overall (n = 217,783)	Delirium (n = 23,100)	Non-delirium (n = 194,683)	Adjusted p value
**Length of hospital stay (in days), mean** ±**SD**	5.4 ± 6.2	10.2 ± 8.2	4.8 ± 5.7	**<0.0001**
**Mortality within 30 days**	7373 (3.4)	2980 (12.9)	4393 (2.3)	**<0.0001**
**Reoperation**	7815 (3.6)	1738 (7.5)	6077 (3.1)	**<0.0001**
**Readmission**	17,843 (8.2)	2606 (11.3)	15,237 (7.8)	**<0.0001**
**Unplanned readmission**	17,538 (8.1)	2578 (11.2)	14,960 (7.7)	**<0.0001**
**Any surgical complication (total patient counts)**	30,345 (13.9)	6191 (26.8)	24,154 (12.4)	**<0.0001**
Superficial incisional infection	4163 (1.9)	589 (2.6)	3574 (1.8)	**<0.0001**
Deep incisional infection	773 (0.4)	137 (0.6)	636 (0.3)	**<0.0001**
Organ space infection	5304 (2.4)	1228 (5.3)	4076 (2.1)	**<0.0001**
Dehiscence	1114 (0.5)	246 (1.1)	868 (0.5)	**<0.0001**
Bleeding/Transfusion	21,652 (9.9)	4717 (20.4)	16,935 (8.7)	**<0.0001**
Postop total transfusion amount, mean ± SD	2.1 ± 2.1	2.3 ± 2.6	2.0 ± 1.9	**<0.0001**
**Any medical complication (total patient counts)**	42,876 (19.7)	23,100 (100.0)	19,776 (10.2)	**<0.0001**
Pneumonia	6998 (3.2)	2679 (11.6)	4319 (2.2)	**<0.0001**
Pulmonary embolism	1677 (0.8)	373 (1.6)	1304 (0.7)	**<0.0001**
Reintubation	2983 (1.4)	1294 (5.6)	1689 (0.7)	**<0.0001**
Ventilator dependence >48 h	3409 (1.6)	1670 (7.2)	1739 (0.9)	**<0.0001**
Postoperative dialysis	1049 (0.5)	436 (1.9)	613 (0.3)	**<0.0001**
Urinary tract infection	6174 (2.8)	1311 (5.7)	4863 (2.5)	**<0.0001**
Cerebral vascular accident/Stroke	1256 (0.6)	455 (2.0)	801 (0.4)	**<0.0001**
Cardiac arrest	1339 (0.6)	446 (1.9)	893 (0.5)	**<0.0001**
Myocardial Infarction	3523 (1.6)	1036 (4.5)	2487 (1.3)	**<0.0001**
Deep vein thrombosis/Thrombophlebitis	2469 (1.1)	592 (2.6)	1877 (1.0)	**<0.0001**
Sepsis	5032 (2.3)	1108 (4.8)	3924 (2.0)	**<0.0001**
Septic shock	4926 (2.3)	1976 (8.6)	2950 (1.5)	**<0.0001**
Clostridium difficile infection	1403 (0.6)	296 (1.3)	1107 (0.6)	**<0.0001**
**Postoperative functional status**				**<0.0001**
Independent	120,735 (59.9)	5081 (27.4)	115,654 (63.2)	
Partially dependent	73,612 (36.5)	10,627 (57.3)	62,985 (34.4)	
Totally dependent	7089 (3.5)	2846 (15.3)	4243 (2.3)	
**Services for home discharge**				**<0.0001**
Discharged to home with services	55,472 (35.5)	4720 (53.3)	50,752 (34.5)	
Discharged to home without services	100,658 (64.5)	4133 (46.7)	96,525 (65.5)	
**Discharge destination**				**<0.0001**
Home/Permanent residence	156,157 (73.1)	8856 (41.7)	147,301 (76.6)	
Acute care	3560 (1.7)	1011 (4.8)	2549 (1.3)	
Against medical advice	152 (0.1)	23 (0.1)	129 (0.2)	
Other facility	49,914 (23.4)	9552 (45.0)	40,362 (22.0)	
Unknown	3732 (1.8)	1779 (8.4)	1953 (1.0)	

SD, standard deviation; h, hours.

Reported as n (%), unless otherwise stated. Significant p values shown in bold. p values adjusted using Bonferroni correction. SD, standard deviation; h, hours.

A slightly higher proportion of delirium patients lived at home with other individuals prior to admission (76.3% vs 73.3%; p < 0.0001), while fewer lived alone (23.7% vs 26.7%; p < 0.0001; Table 1; Fig. 3).

Table 3 compares preoperative laboratory values between patients who developed post-operative delirium and those who did not. All preop variables except PTT showed statistically significant, but mostly clinically irrelevant differences (p < 0.0001) (Table 3). Patients who developed post-operative delirium also experienced longer time from admission to surgery (1.8 ± 4.6 vs 0.8 ± 2.7 days; p < 0.0001).

**Table 3:** Multivariate logistic regression outcomes with respect to the independent predictor delirium.

Outcome variable	Estimator/OR	Adjusted 95% CI	Adjusted p value
Operative time (in minutes)	1.0023	[1.0021, 1.0026]	**<0.0001**
Ventilator dependence >48 h	5.81	[5.09, 6.63]	**<0.0001**
Reintubation	5.35	[4.68, 6.13]	**<0.0001**
Septic shock	4.03	[3.59, 4.53]	**<0.0001**
Pneumonia	3.64	[3.31, 4.01]	**<0.0001**
Mortality within 30 days	3.22	[2.93, 3.54]	**<0.0001**
Length of hospital stay (in days)	3.07	[2.94, 3.20]	**<0.0001**
Organ space infection	2.44	[2.17, 2.76]	**<0.0001**
Myocardial infarction	2.35	[2.04, 2.71]	**<0.0001**
Reoperation	2.28	[2.06, 2.52]	**<0.0001**
Deep vein thrombosis/Thrombophlebitis	2.20	[1.85, 2.61]	**<0.0001**
Urinary tract infection	1.88	[1.67, 2.11]	**<0.0001**
Any surgical complication	1.75	[1.65, 1.86]	**<0.0001**
Bleeding/Transfusion	1.64	[1.54, 1.76]	**<0.0001**
Sepsis	1.62	[1.42, 1.84]	**<0.0001**
Loss of independence	1.61	[1.52, 1.71]	**<0.0001**
Unplanned readmission	1.30	[1.20, 1.41]	**<0.0001**
Superficial incisional infection	1.28	[1.09, 1.51]	**0.0001**
Home discharge	0.48	[0.45, 0.51]	**<0.0001**

OR, odds ratio; CI, confidence interval; h, hours.

Significant p values shown in bold. In the case of operative time, the operative time was used as a predictor, with the outcome delirium. p values adjusted using Bonferroni correction.

### Outcomes associated with delirium

Patients who developed post-operative delirium had significantly worse outcomes and substantially prolonged hospital stays (10 ± 8 vs 5 ± 6 days; p < 0.0001). Delirium was associated with markedly higher rates of reoperation (7.5% vs 3.1%; p < 0.0001), readmission (11.3% vs 7.8%; p < 0.0001), and unplanned readmission (11.2% vs 7.7%; p < 0.0001). Surgical and medical complications were more frequent in the delirium group.

Functionally, delirium patients were more likely to be discharged with partial (57.3% vs 34.4%; p < 0.0001) or total dependence (12.3% vs 2.3%; p < 0.0001). Upon discharge, a substantially larger proportion of patients who experienced delirium required home health services (53.3% vs 34.5%; p < 0.0001). Only 41.7% of delirium patients were discharged to their home or permanent residence compared to 76.6% of non-delirium patients (p < 0.0001).

### Multivariable assessment

On multivariate regression assessment, delirium was associated with a more than three fold increase in 30-day mortality, along with elevated odds of reoperation, unplanned readmission, and any surgical complication. Post-operative delirium was independently associated with a longer hospital stay. Specific surgical complications with increased odds included superficial incisional infection, organ space infection, and bleeding/transfusion, all statistically significant. Delirium was strongly associated with pulmonary complications, specifically increased odds of pneumonia, reintubation, and ventilator dependence >48 h. Additional risks included urinary tract infection, myocardial infarction, deep vein thrombosis/thrombophlebitis, sepsis, and septic shock. Functionally, delirium was associated with increased odds of post-operative loss of independence and a reduced likelihood of home discharge.

## Discussion

This large-scale retrospective analysis of data collected for the NSQIP demonstrates that post-operative delirium is strongly associated with adverse clinical outcomes, significantly impaired functional recovery, and increased health system utilization. Across a total of nearly three million surgical patients, those who were assessed and confirmed to have experienced post-operative delirium markedly differed from patients with no delirium in terms of demographics, comorbidities, surgical characteristics, laboratory profiles, and post-operative trajectories. The findings underscore the importance of delirium as not merely a transient cognitive disturbance but as a critical clinical event linked to systemic dysfunction, longer hospitalization, and long-term disability.

According to NICE Clinical Guideline CG103, all adults aged 65 and older should be screened for delirium within 48 h of emergency hospital admission, although no specific guidelines exist for surgical care.^21^ At the same time, the American Psychiatric Association Practice Guideline for the Prevention and Treatment of Delirium recognizes advancing age (defined as ≥65 years) as a predisposing/contributing factor for delirium.^22^ Our analysis revealed a substantial deviation from this standard. Of the entire surgical population, only 31.2% were screened for delirium, and among those aged 65 and above—who should absolutely have been screened—only 23.4% received appropriate screening. In the entire dataset, only two patients aged between 60 and 70 were screened. This mirrors prior concerns raised by the Geriatric Medicine Research Collaborative in the United Kingdom, whose 2019 World Delirium Day data reported a screening rate of just 27%.^23^ Strikingly, 931,599 patients aged 65+ who met guideline age criteria were not screened at all, highlighting a significant gap between clinical recommendations and real-world practice. Similarly, although the NICE Clinical Guidelines state that presence of cognitive impairment (past or present) and/or dementia, should be confirmed with a standardized and validated cognitive impairment measure, only 56.7% of patients with dementia were screened for delirium.

One of the most pronounced differences between the delirium and non-delirium groups was age. This aligns with prior studies identifying older age as one of the strongest predictors of delirium due to age-related changes in brain function, increased comorbidity burden, and reduced physiological reserve.^24^, ^25^, ^26^, ^27^, ^28^, ^29^, ^30^, ^31^, ^32^, ^33^ Additionally, delirium was more common in those with greater functional dependency. Patients who had experienced a fall in the previous six months or had baseline dementia were also more likely to experience delirium, confirming the role of cognitive and physical frailty as major risk factors.^34^ Patients with delirium also had a significantly higher burden of comorbidities. Notably, the ASA physical status classification was markedly skewed toward higher severity among delirium patients, with over 80% classified as ASA Class 3 or higher, compared to under 50% in non-delirium patients. This supports the interpretation of delirium as a marker of systemic vulnerability. Interestingly, higher BMI did not necessarily predispose to delirium, and may in some cases reflect better physiological reserve. Screened patients were more likely to be diagnosed with delirium in the underweight and normal weight categories. This suggests that underweight or low-normal BMI may be more strongly associated with delirium, possibly reflecting frailty or poor nutritional reserve. It should be noted that a higher proportion of unscreened patients was seen in the overweight and Class I Obesity groups, suggesting that patients with higher BMI were less likely to be screened, implying a possible bias in screening practices (Supplement: Limitations). While obesity is associated with many comorbidities, low BMI is more often linked with frailty, which the literature has previously identified as an independent risk factor for delirium.^35^ Hypoalbuminemia, a marker of malnutrition and inflammation, and inflammatory markers such as elevated WBC count and liver enzymes were also more common in patients who experienced delirium. Zhang et al. studied elderly patients (age ≥65 years) who were admitted to the ICU after non-cardiac surgery and found that severe preoperative hypoalbuminemia significantly increases the risk of delirium.^36^ Hematologic abnormalities, including anemia and mild thrombocytopenia, were also statistically significant. A meta-analysis further highlighted that although preoperative anemia was not always a strong independent predictor, its presence with other metabolic stressors (like hypoalbuminemia) elevated delirium risk.^37^ Our findings reinforce that delirium frequently occurs in patients already exhibiting signs of metabolic stress and end-organ dysfunction prior to surgery.

Urgent and emergency surgeries accounted for a higher proportion of cases in the delirium group, likely due to reduced opportunities for preoperative optimization and increased physiological burden.^8^^,^^38^ Orthopedic, cardiac, and vascular surgeries were disproportionately represented among delirium patients.^39^, ^40^, ^41^ The higher incidence of delirium observed in orthopedic, cardiac, and vascular patients in our study is consistent with prior literature and likely reflects a convergence of risk factors unique to these populations. In orthopedic surgery, particularly after hip fracture repair, delirium risk is heightened by acute pain, immobility, blood loss, anemia, and frequent opioid exposure.^42^ Cardiac surgery patients face additional vulnerabilities including cardiopulmonary bypass–related microemboli, systemic inflammation, and perioperative hemodynamic instability, all of which can impair cerebral perfusion.^43^ Although the NSQIP dataset does not provide mechanistic variables such as intraoperative anesthetic depth, detailed hemodynamics, or biomarkers of neuroinflammation, our findings align with the multifactorial pathophysiology of delirium described in these settings. Future studies incorporating granular perioperative and physiological data will be essential to better delineate the biological pathways underlying delirium in these surgical subgroups. Type of anesthesia was also relevant. General anesthesia remained the predominant modality in both groups, with its relative frequency higher among delirium patients, who were less likely to receive spinal or monitored anesthesia. Prior studies failed to find a link between general anesthesia and postoperative delirium.^44^^,^^45^ Delirium was predominantly an inpatient phenomenon, likely due to longer hospital stays, more invasive procedures, and higher medical complexity. Longer surgeries were significantly and independently associated with an increased risk of postoperative delirium, with the odds increasing by 0.2% for every additional minute of operative time. This effect, though small per minute, accumulates with longer cases—for example, a 100-min longer surgery increases odds by roughly 26%. This adjusted effect size was substantially larger than that observed in the univariable analysis, indicating that longer procedures may not just correlate with but potentially contribute to the development of delirium. Together, these findings support the hypothesis that prolonged surgical duration is not merely associated with delirium but is likely a significant contributing factor, possibly due to extended anesthesia exposure, increased physiological stress, or surgical complexity.

The perioperative trajectory for delirium patients was markedly worse. They experienced longer operative times, longer time from admission to operation, and longer hospital stays. Reoperation, readmission, and unplanned readmission were significantly more frequent. Delirium patients also had significantly higher rates of surgical and medical complications. These results support the interpretation of delirium as both a consequence and driver of systemic complications. Prior research has shown that critically ill surgical patients who develop delirium have significantly more complications such as pneumonia, sepsis, stroke, and myocardial infarction, in addition to extended ventilator support.^46^ Another large-scale study found that delirium during an ICU stay after major surgery predicted worse outcomes, mortality and reduced quality of life.^47^ Multivariate regression confirmed that delirium independently predicts worse outcomes, even after adjusting for confounders. These adjusted models confirm that delirium is not simply a surrogate for age or comorbidity but is a potent, independent predictor of adverse outcomes. Delirium significantly impacted discharge planning and long-term independence. While the majority of non-delirium patients were discharged home without services, delirium patients were much more likely to require home health services or specialized facility placement. This difference emphasizes the long-lasting functional consequences of post-operative delirium. Patients who experience postoperative delirium often require post-acute care such as home health or skilled nursing facilities, and they experience persistent declines in functional status. A large cohort study found that patients with delirium had much higher rates of institutional discharge and functional dependence.^41^

The limitations of this study should be acknowledged, and the findings interpreted within the context of the constraints of the NSQIP database. As with any large registry study based on routinely collected clinical data, there is inherent variability in the accuracy and completeness of the information recorded. Although the NSQIP employs standardized definitions and data collection protocols, the accuracy of the dataset depends heavily on site-level factors such as personnel training, case volume, and institutional resources. One limitation of our analysis is the possibility that delirium screening was performed but not documented, particularly if the screening was uneventful and yielded negative results. In addition, discharge disposition variables in the NSQIP are subject to potential misclassification. For instance, patients residing in assisted living facilities may be coded as “home/permanent residence” on admission but as “other facility” on discharge, creating the appearance of a transition in living setting where none occurred. Such variability likely reflects differences in documentation practices rather than true changes in discharge status. This source of misclassification bias should be considered when interpreting discharge destination results, particularly visualizations such as the Sankey diagram. Furthermore, NSQIP data are primarily drawn from large academic medical centers, which may limit the generalizability of the findings to smaller community hospitals or underserved healthcare settings. A central limitation in this study relates to selection bias. We found that most patients in the NSQIP database were not assessed for delirium, while the identification of post-operative delirium relies on documentation rather than standardized screening tools like the CAM. The reliability of a study's conclusions depends fundamentally on the validity and precision of the metric used to define and assess its primary outcome. Although postoperative delirium was marked as present when delirium was present on screening no details are provided on when delirium occurred after surgery, the screening tools used, the frequency of screening, the postoperative day of screening. This restricts our ability to evaluate temporal patterns or duration of postoperative delirium, while the process of screening affects the rates of diagnosis of delirium.^48^ As delirium screening was not standardized across centers, different tools may have been applied in practice, each with differing sensitivity and specificity. This variability introduces the possibility of both underestimation and overestimation of delirium incidence. Nonetheless, the consistent and strong associations we observed between delirium and adverse outcomes across a large and heterogeneous patient population suggest that the prognostic significance of delirium is robust despite these methodological differences. These findings underscore the urgent need for standardized, validated screening protocols to improve accuracy, comparability, and equity in perioperative delirium assessment. As a result, delirium may be underdiagnosed, particularly among populations less likely to receive formal cognitive evaluation, such as women, Black patients, and those of Hispanic ethnicity. This likely contributes to the racial and ethnic disparities observed in our population and underscores the need for standardized delirium screening protocols across all demographics. We acknowledge that the NSQIP database does not provide information on the temporal sequence of delirium relative to perioperative complications, and therefore temporality between delirium and associated complications cannot be established. Selection bias is also present, for example delirium predominantly occurred in inpatient procedures, but it is possible that patients selected for inpatient procedures were also patients assessed to be more at risk for complications. Another important limitation is related to missing data, particularly with respect to perioperative creatinine measurements. Only patients from the years 2022 and 2023 had data available on perioperative changes in creatinine. This restriction introduces a potential selection bias because the overall cohort spans more years (2021–2023), and delirium incidence was seen to vary over time possibly due to evolving institutional practices, screening frequency, or heightened clinical awareness. If the risk of delirium or its documentation was influenced by the year of surgery, then excluding earlier data could affect the representativeness and comparability of this subgroup analysis. While many relevant preoperative and intraoperative variables were available, the NSQIP dataset lacks data in some clinically important areas. For example, while the use of corticosteroids and immunosuppressants is documented, no information is available regarding drug dosage, duration, or specific indications, limiting the ability to assess dose–response relationships. Similarly, the NSQIP does not collect data on preoperative delirium screening or detailed social determinants of health such as caregiver support, education level, or language barriers—all of which may play critical roles in both delirium risk and recovery but remain unmeasured confounders in this analysis. The NSQIP also only tracks outcomes up to 30 days post-surgery, which precludes assessment of longer-term complications or persistent functional decline often associated with delirium. Outcomes such as readmissions occurring after prolonged hospital stays or delayed institutionalization are not captured, potentially underestimating the full burden of post-operative delirium. Finally, due to the observational nature of this study, causality cannot be definitively established. While multivariate analyses were performed to adjust for known confounders, residual confounding due to unmeasured variables remains possible. Despite these limitations, the NSQIP database remains one of the most robust, validated, and widely used surgical registries,^49^ offering a valuable lens through which to examine patterns of post-operative complications such as delirium at a multicentric scale.

This large-scale analysis reinforces that postoperative delirium is a significant and under-recognized complication associated with a wide range of adverse outcomes. Its pervasive impact across the perioperative continuum highlights the urgent need for proactive, multidisciplinary prevention strategies. Key measures include preoperative risk stratification—particularly for frailty, cognitive impairment, and recent falls—perioperative optimization, and postoperative monitoring and early intervention. From a systems perspective, delirium should be treated as a major perioperative event with implications for quality metrics, discharge planning, and health resource allocation. Future research should focus on implementing and evaluating targeted prevention protocols in high-risk surgical cohorts.

## Contributors

ACP and GH conceived the study, developed the methodology, conducted the statistical analysis and data visualization, and drafted the original manuscript. ACP additionally led data collection efforts. SF, JR, TS, and TN contributed to data collection, methodology, statistical analysis, and drafting of the original manuscript. LK contributed to data collection and critically revised the manuscript. SK, ST, LH, MH, CR, and DPO provided critical revisions and input during manuscript development. All authors reviewed and approved the final manuscript for submission. ACP and GH accessed and verified the underlying data.

## Data sharing statement

The data that support the findings of this study are derived from the American College of Surgeons National Surgical Quality Improvement Program (ACS NSQIP). The ACS NSQIP Participant Use Data Files are available to researchers through application to the ACS NSQIP. Per ACS policy, these data cannot be shared publicly by the authors. Analysis code and related materials are available from the corresponding author upon reasonable request.

## Declaration of interests

All authors have nothing to disclose.
