# Supplementary material for: Delirium after surgery: a retrospective study of predictors, complications, and screening patterns in the national surgical quality improvement program
Source: eClinicalMedicine. 2025 Nov 7;90:103629. doi: 10.1016/j.eclinm.2025.103629 (PMC12766415; doi:10.1016/j.eclinm.2025.103629)
Supplement: Supplement [file mmc1.docx]

**Supplement**

**Supplemental Table 1. Perioperative variables extracted from the NSQIP database.**

| **Variables** |
| --- |
| **Preoperative data** |
| Age (years)  Body mass index (BMI; kg/m²) (calculated from Height in inches and Weight in pounds)  Gender (self-identified: female, male, non-binary)  Race (self-identified: American Indian/Alaskan Native, Asian, Native Hawaiian/other Pacific Islander, Black/African American, White, Other or combinations with low frequency, Unknown)  Ethnicity (Hispanic, non-Hispanic)  Obesity (calculated BMI>30 kg/m²)  Comorbidities [Diabetes (insulin-treated or oral medication), Chronic obstructive pulmonary disease/COPD, Congestive heart failure/CHF, Ascites, Dialysis, Bleeding disorders, Preoperative transfusion, Preoperative sepsis, Ventilator dependence, Hypertension, Smoking status, Disseminated cancer, Corticosteroid use, Dementia, History of a fall within the last six months)  Immunosuppressive therapy (Corticosteroids, Anti-rejection/transplant immunosuppressants, Synthetic DMARDs/DMDs, Biologic DMARDs/DMDs, Other)  American Society of Anesthesiologists (ASA) Class (1 – No disturbance, 2 – Mild disturbance, 3 – Severe disturbance, 4 – Life-threatening, 5 – Moribund)  Origin location (Home/Permanent residence, Acute area hospital, Other facility, Unknown)  Preoperative functional status (independent, partially or totally dependent)  Most severe preoperative creatinine increase  Home support (Lives at home with other individuals, Lives alone at home) |
| **Surgical characteristics** |
| Urgency (elective and urgent/emergent surgeries)  Specialty (cardiac, general, gynecology, neurosurgery, orthopedics, otolaryngology, plastics, thoracic, urology, vascular, obstetrics, interventional radiology)  Anesthesia (general, monitored anesthesia care, epidural, local, regional, spinal, none, other)  Setting (inpatient or outpatient)  Year (2021, 2022 or 2023)  Pre-operative lab values (Serum sodium, Blood urea nitrogen/BUN, Creatinine, Serum albumin, Total bilirubin, Serum Glutamic-Oxaloacetic Transaminase, also known as Aspartate Aminotransferase/SGOT, Alkaline Phosphatase/ALP, white blood cells/WBC, Hematocrit, platelet count, Partial Thromboplastin Time/PTT, International Normalized Ratio/INR, Hemoglobin A1c/HbA1c) |
| **Outcomes** |
| Time from admission to operation (days)  Operative time (minutes)  Length of hospital stay (days; difference in days between the date of admission and the date of discharge)  Mortality  Reoperation  (Unplanned) readmission  Surgical complications (superficial/deep incision site/organ space infection, Dehiscence, Bleeding/transfusion, Postop total transfusion amount)  Medical complications (Pneumonia, Pulmonary embolism, Reintubation, Ventilator dependence >48 hours, Postoperative dialysis, Urinary tract infection, Cerebral vascular accidents/strokes, Cardiac arrest, Myocardial infarction, Deep vein thrombosis/thrombophlebitis, Sepsis, Septic shock, Clostridium difficile infection, Delirium  Post-operative functional status (independent, partially or totally dependent)  Most severe postoperative creatinine increase  Discharge destination (Home/permanent residence), Acute care, Against medical advice, Other, Unknown)  Services for home discharge (Discharged to home with services, Discharged to home without services) |

**Supplemental Table 2. Patient demographics and comorbidities.** Patients grouped according to delirium. Reported as n (%), unless otherwise stated. Significant p values shown in bold. p values adjusted using Bonferroni correction.

|  | | **Overall**  **(n = 2,990,064)** | | **Group 1+2**  **(n = 217,783)** | | **Unscreened**  **(n = 2,772,281)** | | **Adjusted**  **p value** | |
| --- | --- | --- | --- | --- | --- | --- | --- | --- | --- |
| **Demographics** | |  | |  | |  | |  | |
| Age, mean ± SD | | 56·5 ± 17·0 | | 80·0 ± 4·0 | | 54·8 ± 16·3 | | **<0·0001** | |
| Age > 90 | | 33,499 (1·1) | | 18,766 (8·6) | | 14,733 (0·5) | | **<0·0001** | |
| BMI (kg/m^2^), mean ± SD | | 30·3 ± 7·2 | | 27·6 ± 5·8 | | 30·5 ± 7·3 | | **<0·0001** | |
| **Gender** | |  | |  | |  | | **<0·0001** | |
| Female | | 1,727,578 (57·8) | | 121,201 (55·7) | | 1,606,377 (57·9) | |  | |
| Male | | 1,260,591 (42·2) | | 96,576 (44·4) | | 1,164,015 (42·0) | |  | |
| Non-binary | | 1,895 (0·1) | | 6 (0·0) | | 1,889 (0·1) | |  | |
| **Race** | |  | |  | |  | | **<0·0001** | |
| American Indian or Alaskan native | | 20,606 (0·7) | | 689 (0·3) | | 19,917 (0·7) | |  | |
| Asian | | 102,215 (3·4) | | 6,606 (3·0) | | 95,609 (3·5) | |  | |
| Native Hawaiian or Pacific Islander | | 10,219 (0·3) | | 338 (0·2) | | 9,881 (0·4) | |  | |
| Black or African American | | 290,897 (9·7) | | 10,770 (5·0) | | 280,127 (10·1) | |  | |
| White | | 1,856,514 (62·1) | | 136,489 (62·7) | | 1,720,025 (62·0) | |  | |
| Other (other race/combinations) | | 75,476 (2·5) | | 2,893 (1·3) | | 72,583 (2·6) | |  | |
| Unknown | | 634,137 (21·2) | | 59,998 (27·6) | | 574,139 (20·7) | |  | |
| **Ethnicity** | |  | |  | |  | |  | |
| Hispanic | | 300,212 (12·4) | | 8,401 (5·3) | | 291,811 (12·9) | | **<0·0001** | |
| **Preoperative health and comorbidities** | |  | |  | |  | |  | |
| Obesity | | 1,306,422 (45·1) | | 60,708 (29·6) | | 1,245,714 (46·3) | | **<0·0001** | |
| Diabetes | | 470,263 (15·7) | | 45,921 (21·1) | | 424,342 (15·3) | | **<0·0001** | |
| Insulin treated | | 151,949 (32·3) | | 14,175 (30·9) | | 137,774 (32·5) | | **<0·0001** | |
| COPD | | 111,185 (3·7) | | 17,623 (8·1) | | 93,562 (3·4) | | **<0·0001** | |
| CHF | | 94,868 (3·2) | | 20,673 (9·5) | | 74,195 (2·7) | | **<0·0001** | |
| Ascites | | 16,069 (0·5) | | 1,956 (0·9) | | 14,113 (0·5) | | **<0·0001** | |
| Dialysis | | 26,706 (0·9) | | 2,407 (1·1) | | 24,299 (0·9) | | **<0·0001** | |
| Bleeding disorder | | 105,647 (3·5) | | 19,023 (8·7) | | 86,624 (3·1) | | **<0·0001** | |
| Transfusion | | 23,699 (0·8) | | 4,282 (2·0) | | 19,417 (0·7) | | **<0·0001** | |
| Pre-operative sepsis | | 183,325 (6·1) | | 19,957 (9·2) | | 163,368 (5·9) | | **<0·0001** | |
| Ventilator dependent | | 8,023 (0·3) | | 762 (0·4) | | 7,261 (0·3) | | **<0·0001** | |
| Hypertension | | 1,283,777 (42·9) | | 156,917 (72·1) | | 1,126,860 (40·7) | | **<0·0001** | |
| Current smoker | | 398,850 (13·3) | | 12,603 (5·8) | | 386,247 (13·9) | | **<0·0001** | |
| Disseminated cancer | | 65,499 (2·2) | | 7,108 (3·3) | | 58,391 (2·1) | | **<0·0001** | |
| Corticosteroid use | | 128,176 (4·3) | | 11,587 (5·3) | | 116,589 (4·2) | | **<0·0001** | |
| Dementia | | 42,083 (8·9) | | 23,842 (11·0) | | 18,241 (7·2) | | **<0·0001** | |
| Fall within last 6 months | | 77,395 (18·1) | | 41,821 (20·7) | | 35,574 (15·8) | | **<0·0001** | |
| Immunosuppressive Therapy | |  | |  | |  | |  | |
| Corticosteroids | | 61,297 (2·1) | | 6,701 (3·1) | | 54,596 (2·0) | | **<0·0001** | |
| Anti-rejection/transplant immunosuppressants | | 18,048 (0·6) | | 962 (0·4) | | 17,086 (0·6) | | **<0·0001** | |
| Synthetic DMARDs/DMDs | | 35,430 (1·2) | | 3,326 (1·5) | | 32,104 (1·2) | | **<0·0001** | |
| Biologic DMARDs/DMDs | | 29,141 (1·0) | | 1,401 (0·6) | | 27,740 (1·0) | | **<0·0001** | |
| Other | | 11,210 (0·4) | | 1,019 (0·5) | | 10,191 (0·4) | | **<0·0001** | |
| **ASA Class** | |  | |  | |  | | **<0·0001** | |
| 1 – No disturbance | | 195,448 (6·6) | | 670 (0·3) | | 194,778 (7·0) | |  | |
| 2 – Mild disturbance | | 1,321,475 (44·3) | | 42,377 (19·5) | | 1,279,098 (46·3) | |  | |
| 3 – Severe disturbance | | 1,292,075 (43·3) | | 137,507 (63·3) | | 1,154,568 (41·8) | |  | |
| 4 – Life-threatening | | 166,905 (5·6) | | 35,585 (16·4) | | 131,320 (4·8) | |  | |
| 5 – Moribund | | 6,239 (0·2) | | 1,094 (0·5) | | 5,145 (0·2) | |  | |
| **Origin Status** | |  | |  | |  | | **<0·0001** | |
| Home/Permanent residence | | 2,874,712 (96·1) | | 200,185 (91·9) | | 2,674,527 (96·5) | |  | |
| Acute care hospital | | 82,753 (2·8) | | 12,363 (5·7) | | 70,390 (2·5) | |  | |
| Other facility | | 19,719 (0·7) | | 4,142 (1·9) | | 15,577 (0·6) | |  | |
| Unknown | | 12,880 (0·4) | | 1,093 (0·5) | | 11,787 (0·4) | |  | |
| **Preoperative Functional Status** | |  | |  | |  | | **<0·0001** | |
| Independent | | 2,884,673 (97·5) | | 192,994 (89·7) | | 2,691,679 (98·1) | |  | |
| Partially Dependent | | 62,215 (2·1) | | 19,394 (9·0) | | 42,821 (1·6) | |  | |
| Totally Dependent | | 11,149 (0·4) | | 2,679 (1·3) | | 8,470 (0·3) | |  | |

**Supplemental Table 3**. **Surgical characteristics**. Patients grouped according to delirium. Reported as n (%). Significant p values shown in bold. p values adjusted using Bonferroni correction.

| **Characteristic** | **Overall**  **(n = 2,990,064)** | **Group 1+2**  **(n = 217,783)** | **Unscreened**  **(n = 2,772,281)** | **Adjusted**  **p value** |
| --- | --- | --- | --- | --- |
| **Urgency** |  |  |  | **<0·0001** |
| Elective | 2,469,839 (82·6) | 155,088 (71·2) | 2,314,751 (83·5) |  |
| Urgent/Emergent | 520,225 (17·4) | 62,695 (28·8) | 457,530 (16·5) |  |
| **Surgical specialty** |  |  |  | **<0·0001** |
| Cardiac | 13,439 (0·5) | 1,480 (0·7) | 11,959 (0·4) |  |
| General Surgery | 1,226,272 (41·0) | 74,140 (34·0) | 1,152,132 (41·6) |  |
| Gynecology | 306,548 (10·3) | 5,843 (2·7) | 300,705 (10·9) |  |
| Neurosurgery | 160,888 (5·4) | 12,152 (5·6) | 148,736 (5·4) |  |
| Orthopedics | 735,262 (24·6) | 84,444 (38·8) | 650,818 (23·5) |  |
| Otolaryngology | 71,656 (2·4) | 2,794 (1·3) | 68,862 (2·5) |  |
| Plastics | 90,613 (3·0) | 896 (0·4) | 89,717 (3·2) |  |
| Thoracic | 37,052 (1·2) | 4,653 (2·1) | 32,399 (1·2) |  |
| Urology | 197,384 (6·6) | 15,645 (7·2) | 181,739 (6·6) |  |
| Vascular | 98,556 (3·3) | 15,638 (7·2) | 82,918 (3·0) |  |
| Obstetrics | 52,075 (1·7) | 68 (0·0) | 52,007 (1·9) |  |
| Interventional Radiology | 319 (0·0) | 30 (0·01) | 289 (0·0) |  |
| **Type of Anesthesia** |  |  |  | **<0·0001** |
| General | 2,597,866 (86·9) | 171,637 (78·8) | 2,426,229 (87·5) |  |
| Monitored anesthesia care | 157,806 (5·3) | 17,257 (7·9) | 140,549 (5·1) |  |
| Epidural | 14,697 (0·5) | 367 (0·2) | 14,330 (0·5) |  |
| Local | 3,416 (0·1) | 229 (0·1) | 3,187 (0·1) |  |
| Regional | 22,721 (0·8) | 2,446 (1·1) | 20,275 (0·7) |  |
| Spinal | 191,529 (6·4) | 25,686 (11·8) | 165,843 (6·0) |  |
| None | 164 (0·01) | 15 (0·0) | 149 (0·0) |  |
| Other | 1,672 (0·1) | 140 (0·1) | 1,532 (0·1) |  |
| **Setting** |  |  |  | **<0·0001** |
| Inpatient | 1,455,477 (48·7) | 169,599 (77·9) | 1,285,878 (46·4) |  |
| Outpatient | 1,534,587 (51·3) | 48,184 (22·1) | 1,486,403 (53·6) |  |
| **Year** |  |  |  | **<0·0001** |
| 2021 | 983,851 (32·9) | 65,822 (30·2) | 918,029 (33·1) |  |
| 2022 | 1,011,899 (33·8) | 72,373 (33·2) | 939,526 (33·9) |  |
| 2023 | 994,314 (33·3) | 79,588 (36·5) | 914,726 (33·0) |  |

**Supplemental Table 4**. **Peri- and postoperative outcomes.** Patients grouped according to delirium. Reported as n (%), unless otherwise stated. Significant p values shown in bold. p values adjusted using Bonferroni correction.

| **Outcomes** | | **Overall**  **(n = 2,990,064)** | | | **Screened**  **(n = 217,783)** | | **Unscreened**  **(n = 2,772,281)** | | **Adjusted**  **p value** | |
| --- | --- | --- | --- | --- | --- | --- | --- | --- | --- | --- |
| **Time from admission to operation (days), mean ± SD** | | 0·5 ± 2·3 | | 0·9 ± 3·0 | | | 0·4 ± 2·2 | | **<0·0001** | |
| **Operative time (in minutes), mean ± SD** | | 116·4 ± 94·8 | | 122·5 ± 94·1 | | | 115·9 ± 94·8 | | **<0·0001** | |
| **Length of Hospital Stay (in days), mean ±SD** | | 2·6 ± 4·6 | | 5·4 ± 6·2 | | | 2·4 ± 4·4 | | **<0·0001** | |
| **Reoperation** | | 71,230 (2·4) | | 7,815 (3·6) | | | 63,415 (2·3) | | **<0·0001** | |
| **Readmission** | | 138,860 (4·7) | | 17,843 (8·2) | | | 121,017 (4·4) | | **<0·0001** | |
| **Unplanned Readmission** | | 134,243 (4·5) | | 17,538 (8·1) | | | 116,705 (4·2) | | **<0·0001** | |
| **Any Surgical Complication (total patient counts)** | | 223,128 (7·5) | | 30,345 (13·9) | | | 192,783 (7·0) | | **<0·0001** | |
| Superficial Incisional Infection | | 52,120 (1·7) | | 4,163 (1·9) | | | 47,957 (1·7) | | **<0·0001** | |
| Deep Incisional Infection | | 9,451 (0·3) | | 773 (0·4) | | | 8,678 (0·3) | | 0·0656 | |
| Organ Space Infection | | 52,868 (1·8) | | 5,304 (2·4) | | | 47,564 (1·7) | | **<0·0001** | |
| Dehiscence | | 11,254 (0·4) | | 1,114 (0·5) | | | 10,140 (0·4) | | **<0·0001** | |
| Bleeding/Transfusion | | 117,418 (3·9) | | 21,652 (9·9) | | | 95,766 (3·5) | | **<0·0001** | |
| Postop total transfusion amount, mean ± SD | | 2·3 ± 2·9 | | 2·06 ± 2·1 | | | 2·34 ± 3·0 | | **<0·0001** | |
| **Any Medical Complication (total patient counts)** | | 176,204 (50·2) | | 42,876 (19·7) | | | 133,328 (100·0) | | **<0·0001** | |
| Pneumonia | | 31,744 (1·1) | | 6,998 (3·2) | | | 24,746 (0·9) | | **<0·0001** | |
| Pulmonary embolism | | 10,392 (0·4) | | 1,677 (0·8) | | | 8,715 (0·3) | | **<0·0001** | |
| Reintubation | | 15,442 (0·5) | | 2,983 (1·4) | | | 12,459 (0·5) | | **<0·0001** | |
| Ventilator Dependence >48h | | 21,972 (0·7) | | 3,409 (1·6) | | | 18,563 (0·7) | | **<0·0001** | |
| Postoperative Dialysis | | 6,472 (0·2) | | 1,049 (0·5) | | | 5,423 (0·2) | | **<0·0001** | |
| Urinary Tract Infection | | 38,386 (1·3) | | 6,174 (2·8) | | | 32,212 (1·2) | | **<0·0001** | |
| Cerebral Vascular Accident/Stroke | | 5,247 (0·2) | | 1,256 (0·6) | | | 3,991 (0·1) | | **<0·0001** | |
| Cardiac Arrest | | 7,551 (0·3) | | 1,339 (0·6) | | | 6,212 (0·2) | | **<0·0001** | |
| Myocardial Infarction | | 11,249 (0·4) | | 3,523 (1·6) | | | 7,726 (0·3) | | **<0·0001** | |
| Deep Vein Thrombosis/Thrombophlebitis | | 16,020 (0·5) | | 2,469 (1·1) | | | 13,551 (0·5) | | **<0·0001** | |
| Sepsis | | 39,980 (1·3) | | 5,032 (2·3) | | | 34,948 (1·3) | | **<0·0001** | |
| Septic Shock | | 26,198 (0·9) | | 4,926 (2·3) | | | 21,272 (0·8) | | **<0·0001** | |
| Clostridium difficile infection | | 7,070 (0·2) | | 1,403 (0·6) | | | 5,667 (0·2) | | **<0·0001** | |
| **Postoperative Functional Status** | |  | |  | | |  | | **<0·0001** | |
| Independent | | 312,361 (68·1) | | 120,735 (59·9) | | | 191,626 (74·4) | |  | |
| Partially Dependent | | 133,898 (29·2) | | 73,612 (36·5) | | | 60,286 (23·4) | |  | |
| Totally Dependent | | 12,769 (2·8) | | 7,089 (3·5) | | | 5,680 (2·2) | |  | |

**Supplemental Table 5. Preoperative lab values.** Units shown in brackets (%). Reported as mean ± SD.

| **Characteristic** | **Overall**  **(n = 2,990,064)** | | **Group 1+2**  **(n = 217,783)** | | **Unscreened**  **(n = 2,772,281)** | | **Reference range** | **Adjusted**  **p-value** |  |
| --- | --- | --- | --- | --- | --- | --- | --- | --- | --- |
| Serum sodium (mmol/L) | 138·7 ± 3·0 | 138·5 ± 3·5 | | 138·8 ± 2·9 | | 135-145 | | **<0·0001** | |
| BUN (mg/dL) | 16·6 ± 9·9 | 21·5 ± 12·0 | | 16·1 ± 9·6 | | 8-25 | | **<0·0001** | |
| Creatinine (g/D) | 1·0 ± 0·8 | 1·1 ± 0·7 | | 1·0 ± 0·8 | | F 0·6-1·8, M 0·8-2·4 | | **<0·0001** | |
| Serum albumin (g/dL) | 4·0 ± 0·6 | 3·8 ± 0·6 | | 4·0 ± 0·6 | | 3·1 – 4·3 | | **<0·0001** | |
| Total bilirubin (mg/dL) | 0·7 ± 0·6 | 0·7 ± 0·7 | | 0·6 ± 0·6 | | 0-1 | | **<0·0001** | |
| SGOT (U/L) | 28·7 ± 38·8 | 27·7 ± 33·7 | | 28·8 ± 39·2 | | F 9-25, M 10-40 | | **<0·0001** | |
| ALP (U/L) | 89·6 ± 54·5 | 91·3 ± 59·5 | | 89·5 ± 54·1 | | F 30-100 | | **<0·0001** | |
| WBC (x 103/mm3) | 8·1 ± 3·7 | 8·4 ± 3·9 | | 8·1 ± 3·6 | | 4·5-11 | | **<0·0001** | |
| Hematocrit (% of RBCs) | 39·8 ± 5·3 | 37·9 ± 5·7 | | 39·9 ± 5·2 | | F 36-46, M 37-49 | | **<0·0001** | |
| Platelet count (x 103/µL) | 259·4 ± 83·6 | 238·9 ± 85·4 | | 261·2 ± 83·2 | | 130-400 | | **<0·0001** | |
| PTT (sec) | 30·5 ± 9·0 | 31·3 ± 10·8 | | 30·4 ± 8·8 | | 25-35 | | **<0·0001** | |
| INR | 1·1 ± 0·3 | 1·1 ± 0·3 | | 1·1 ± 0·3 | | <1·1 | | **<0·0001** | |
| HbA1c (%) | 6·2 ± 1·7 | 6·2 ± 1·4 | | 6·2 ± 1·7 | | 4-5·7% | | 1·0000 | |

**Supplemental Table 6.** **Age distribution.** Reported as n (%), where the % is calculated from the total within that age group. For example, of a total of 236,001 75 to 79 year olds, 132,872 (56·3%) were unscreened. Of those screened, 3·1% had delirium.

|  |  |  | | **Screened (n = 217,783)** | | | |
| --- | --- | --- | --- | --- | --- | --- | --- |
| **Characteristic** | **Total**  **(n = 2,990,064)** | **Unscreened**  **(n = 2,772,281)** | | **Delirium**  **(n = 23,100)** | | **No Delirium**  **(n = 194,683)** | |
| **Age** |  |  |  | |  | |  |
| 18-19 | 26,111 | 26,111 (100) | 0 (0·0) | | 0 (0·0) | |  |
| 20-24 | 93,342 | 93,342 (100) | 0 (0·0) | | 0 (0·0) | |  |
| 25-29 | 121,805 | 121,805 (100) | 0 (0·0) | | 0 (0·0) | |  |
| 30-34 | 157,750 | 157,750 (100) | 0 (0·0) | | 0 (0·0) | |  |
| 35-39 | 179,867 | 179,867 (100) | 0 (0·0) | | 0 (0·0) | |  |
| 40-44 | 198,920 | 198,919 (100) | 0 (0·0) | | 1 (0·0) | |  |
| 45-49 | 206,950 | 206,949 (100) | 1 (0·0) | | 0 (0·0) | |  |
| 50-54 | 239,142 | 239,141 (100) | 0 (0·0) | | 1 (0·0) | |  |
| 55-59 | 279,489 | 279,489 (100) | 0 (0·0) | | 0 (0·0) | |  |
| 60-64 | 337,309 | 337,309 (100) | 0 (0·0) | | 0 (0·0) | |  |
| 65-69 | 357,787 | 357,786 (100) | 0 (0·0) | | 1 (0·0) | |  |
| 70-74 | 319,220 | 319,219 (100) | 0 (0·0) | | 1 (0·0) | |  |
| 75-79 | 236,001 | 132,872 (56·3) | 7,428 (3·1) | | 95,701 (40·6) | |  |
| 80-84 | 135,661 | 72,985 (53·8) | 6,355 (4·7) | | 56,321 (41·5) | |  |
| 85-89 | 67,211 | 34,004 (50·6) | 5,036 (7·5) | | 28,171 (41·9) | |  |
| 90+ | 33,499 | 14,733 (44·0) | 4,280 (12·8) | | 14,486 (43·2) | |  |

**Supplemental Table 7.** **Unadjusted multivariate logistic regression outcomes with respect to the independent predictor delirium.** Significant p values shown in bold. In the case of operative time, the operative time was used as a predictor, with the outcome delirium. p values adjusted using Bonferroni correction.

| **Outcome Variable** | **Estimator / OR** | **Adjusted 95% CI** | **Adjusted p value** |
| --- | --- | --- | --- |
| Operative time (in minutes) | 1·0003 | [1·0001, 1·0005] | **0·0007** |
| Length of Hospital Stay (in days) | 5·36 | [5·23, 5·49] | **<0·0001** |
| Mortality within 30 days | 6·42 | [5·95, 6·91] | **<0·0001** |
| Reoperation | 2·53 | [2·32, 2·75] | **<0·0001** |
| Unplanned readmission | 1·51 | [1·41, 1·62] | **<0·0001** |
| Any surgical complication | 2·58 | [2·46, 2·72] | **<0·0001** |
| Superficial Incisional Infection | 1·40 | [1·22, 1·60] | **0·0001** |
| Organ Space Infection | 2·63 | [2·38, 2·90] | **<0·0001** |
| Bleeding/Transfusion | 2·69 | [2·55, 2·84] | **<0·0001** |
| Pneumonia | 5·78 | [5·35, 6·25] | **<0·0001** |
| Reintubation | 6·78 | [6·06, 7·59] | **<0·0001** |
| Ventilator Dependence >48h | 8·65 | [7·78, 9·61] | **<0·0001** |
| Urinary Tract Infection | 2·35 | [2·13, 2·59] | **<0·0001** |
| Myocardial Infarction | 3·63 | [3·24, 4·06] | **<0·0001** |
| Deep Vein Thrombosis/Thrombophlebitis | 2·70 | [2·34, 3·12] | **<0·0001** |
| Sepsis | 2·45 | [2·21, 2·72] | **<0·0001** |
| Septic Shock | 6·08 | [5·56, 6·65] | **<0·0001** |
| Loss of independence | 2·33 | [2·23, 2·44] | **<0·0001** |
| Home discharge | 0·24 | [0·23, 0·26] | **<0·0001** |

OR, odds ratio; CI, confidence interval; h, hours.

**Supplemental Table 8.** **Comparison of patient characteristics between the delirium and non-delirium groups.** Reported as n (% of those screened for delirium).

| **Characteristic** | **Delirium**  **(n = 23,100; 10·61%)** | **Non-delirium**  **(n = 194,683; 89·39%)** |
| --- | --- | --- |
| **Demographics** |  |  |
| Age <= 90 | 18,820 (9·5) | 180,197 (90·5) |
| Age > 90 | 4,280 (22·8) | 14,486 (77·2) |
| **Gender** |  |  |
| Female | 12,865 (10·6) | 108,336 (89·4) |
| Male | 10,233 (10·6) | 86,343 (89·4) |
| Non-binary | 2 (33·3) | 4 (66·7) |
| **Race** |  |  |
| American Indian or Alaskan native | 83 (12·1) | 606 (88·0) |
| Asian | 604 (9·1) | 6,002 (90·9) |
| Native Hawaiian or Pacific Islander | 32 (9·5) | 306 (90·5) |
| Black or African American | 1,204 (11·2) | 9,566 (88·8) |
| White | 12,743 (9·3) | 123,746 (90·7) |
| Other (other race/combinations) | 279 (9·6) | 2,614 (90·4) |
| Unknown | 8,155 (13·6) | 51,843 (86·4) |
| **Ethnicity** |  |  |
| Non-Hispanic | 14,423 (9·5) | 137,100 (90·5) |
| Hispanic | 861 (10·3) | 7,540 (89·8) |
| **ASA Class** |  |  |
| 1 – No disturbance | 26 (3·9) | 644 (96·1) |
| 2 – Mild disturbance | 1,923 (4·5) | 40,454 (95·5) |
| 3 – Severe disturbance | 13,757 (10·0) | 123,750 (90·0) |
| 4 – Life-threatening | 6,925 (19·5) | 28,660 (80·5) |
| 5 – Moribund | 379 (34·6) | 715 (65·4) |
| **Preoperative health and comorbidities** |  |  |
| Obesity: No | 15,545 (10·8) | 128,729 (89·2) |
| Yes | 4,679 (7·7) | 56,029 (92·3) |
| Diabetes: No | 17,941 (10·4) | 153,921 (89·6) |
| Yes | 5,159 (11·2) | 40,762 (88·8) |
| Insulin treated: No | 3,261 (10·3) | 28,485 (89·7) |
| Yes | 1,898 (13·4) | 12,277 (86·6) |
| COPD: No | 20,558 (10·3) | 179,602 (89·7) |
| Yes | 2,542 (14·4) | 15,081 (85·6) |
| CHF: No | 19,771 (10·0) | 177,339 (90·0) |
| Yes | 3,329 (16·1) | 17,344 (83·9) |
| Ascites: No | 22,662 (10·5) | 193,165 (89·5) |
| Yes | 438 (22·4) | 1,518 (77·6) |
| Dialysis: No | 22,651 (10·5) | 192,725 (89·5) |
| Yes | 449 (18·7) | 1,958 (81·4) |
| Bleeding disorder: No | 19,933 (10·0) | 178,827 (90·0) |
| Yes | 3,167 (16·7) | 15,856 (83·4) |
| Transfusion: No | 22,099 (10·4) | 191,402 (89·7) |
| Yes | 1,001 (23·4) | 3,281 (76·6) |
| Pre-operative sepsis: No | 18,643 (9·4) | 179,183 (90·6) |
| Yes | 4,457 (22·3) | 15,500 (77·7) |
| Ventilator dependent: No | 22,778 (10·5) | 194,243 (89·5) |
| Yes | 322 (42·3) | 440 (57·7) |
| Hypertension: No | 6,509 (10·7) | 54,357 (89·3) |
| Yes | 16,591 (10·6) | 140,326 (89·4) |
| Current smoker: No | 21,372 (10·4) | 183,808 (89·6) |
| Yes | 1,728 (13·7) | 10,875 (86·3) |
| Disseminated cancer: No | 22,185 (10·5) | 188,490 (89·5) |
| Yes | 915 (12·9) | 6,193 (87·1) |
| Corticosteroid use: No | 21,781 (10·6) | 184,415 (89·4) |
| Yes | 1,319 (11·4) | 10,268 (88·6) |
| Dementia: No | 14,547 (7·5) | 179,394 (92·5) |
| Yes | 8,553 (35·9) | 15,289 (64·1) |
| Fall within last 6 months: No | 12,809 (8·0) | 147,538 (92·0) |
| Yes | 8,621 (20·6) | 33,200 (79·4) |
| Immunosuppressive Therapy |  |  |
| Corticosteroids: No | 22,190 (10·5) | 188,892 (89·5) |
| Yes | 910 (13·6) | 5,791 (86·4) |
| Anti-rejection/transplant immunosuppressants: No | 22,972 (10·6) | 193,849 (89·4) |
| Yes | 128 (13·3) | 834 (86·7) |
| Synthetic DMARDs/DMDs: No | 22,847 (10·7) | 191,610 (89·4) |
| Yes | 253 (7·6) | 3,073 (92·4) |
| Biologic DMARDs/DMDs: No | 22,981 (10·6) | 193,401 (89·4) |
| Yes | 119 (8·5) | 1,282 (91·5) |
| Other: No | 22,986 (10·6) | 193,778 (89·4) |
| Yes | 114 (11·2) | 905 (88·8) |
| **Origin Location** |  |  |
| Home/Permanent residence | 19,118 (9·6) | 181,067 (90·5) |
| Acute care hospital | 2,731 (22·1) | 9,632 (77·9) |
| Other facility | 1,184 (28·6) | 2,958 (71·4) |
| Unknown | 67 (6·1) | 1,026 (93·9) |
| **Preoperative Functional Status** |  |  |
| Independent | 16,474 (8·5) | 176,520 (91·5) |
| Partially Dependent | 5,159 (26·6) | 14,235 (73·4) |
| Totally Dependent | 1,098 (41·0) | 1,581 (59·0) |
| **Home support** |  |  |
| Lives at home with other individuals | 14,089 (10·1) | 125,987 (89·9) |
| Lives alone at home | 4,373 (8·7) | 45,902 (91·3) |
| **Surgical urgency** |  |  |
| Elective | 10,468 (6·8) | 144,620 (93·3) |
| Urgent/Emergency | 12,632 (20·2) | 50,063 (79·9) |
| **Surgical specialty** |  |  |
| Cardiac | 266 (18·0) | 1,214 (82·0) |
| General Surgery | 7,703 (10·4) | 66,437 (89·6) |
| Gynecology | 287 (4·9) | 5,556 (95·1) |
| Neurosurgery | 1,182 (9·7) | 10,970 (90·3) |
| Orthopedics | 10,685 (12·7) | 73,759 (87·4) |
| Otolaryngology | 171 (6·1) | 2,623 (93·9) |
| Plastics | 62 (6·9) | 834 (93·1) |
| Thoracic | 301 (6·5) | 4,352 (93·5) |
| Urology | 898 (5·7) | 14,747 (94·3) |
| Vascular | 1,537 (9·8) | 14,101 (90·2) |
| Obstetrics | 2 (2·9) | 66 (97·1) |
| Interventional Radiology | 6 (20·0) | 24 (80·0) |
| **Type of Anesthesia** |  |  |
| General | 18,810 (11·0) | 152,827 (89·0) |
| Monitored anesthesia care | 1,529 (8·9) | 15,728 (91·1) |
| Epidural | 30 (8·2) | 337 (91·8) |
| Local | 19 (8·3) | 210 (91·7) |
| Regional | 185 (7·6) | 2,261 (92·4) |
| Spinal | 2,510 (9·8) | 23,176 (90·2) |
| None | 4 (26·7) | 11 (73·3) |
| Other | 13 (9·3) | 127 (90·7) |
| **Operative setting** |  |  |
| Inpatient | 21,577 (12·7) | 148,022 (87·3) |
| Outpatient | 1,523 (3·7) | 46,661 (96·8) |
| **Year** |  |  |
| 2021 | 8,487 (12·9) | 57,335 (87·1) |
| 2022 | 7,676 (10·6) | 64,697 (89·4) |
| 2023 | 6,937 (8·7) | 72,651 (91·3) |
| **Mortality within 30 days: No** | 20,120 (9·6) | 190,290 (90·4) |
| **Yes** | 2,980 (40·4) | 4,393 (59·6) |
| **Reoperation: No** | 21,362 (10·2) | 188,606 (89·8) |
| **Yes** | 1,738 (22·24) | 6,077 (77·76) |
| **Readmission: No** | 20,494 (10·25) | 179,446 (89·75) |
| **Yes** | 2,606 (14·61) | 15,237 (85·39) |
| **Unplanned Readmission: No** | 20,522 (10·25) | 179,723 (89·75) |
| **Yes** | 2,578 (14·70) | 14,960 (85·30) |
| **Any Surgical Complication: No (total patient counts)** | 16,909 (9·02) | 170,529 (90·98) |
| **Yes** | 6,191 (20·40) | 24,154 (79·60) |
| Superficial Incisional Infection: No | 22,511 (10·54) | 191,109 (89·46) |
| Yes | 589 (14·15) | 3,574 (85·85) |
| Deep Incisional Infection: No | 22,963 (10·58) | 194,047 (89·42) |
| Yes | 137 (17·72) | 636 (82·28) |
| Organ Space Infection: No | 21,872 (10·29) | 190,607 (89·71) |
| Yes | 1,228 (23·15) | 4,076 (76·85) |
| Dehiscence: No | 22,854 (10·55) | 193,815 (89·45) |
| Yes | 246 (22·08) | 868 (77·92) |
| Bleeding/Transfusion: No | 18,383 (9·37) | 177,748 (90·63) |
| Yes | 4,717 (21·79) | 16,935 (78·21) |
| **Any Medical Complication: No (total patient counts)** | 0 (0·00) | 174,907 (100·00) |
| **Yes** | 23,100 (53·88) | 19,776 (46·12) |
| Pneumonia: No | 20,421 (9·69) | 190,364 (90·31) |
| Yes | 2,679 (38·28) | 4,319 (61·72) |
| Pulmonary embolism: No | 22,727 (10·52) | 193,379 (89·48) |
| Yes | 373 (22·24) | 1,304 (77·76) |
| Reintubation: No | 21,806 (10·15) | 192,994 (89·85) |
| Yes | 1,294 (43·38) | 1,689 (56·62) |
| Ventilator Dependence >48h: No | 21,430 (10·00) | 192,944 (90·00) |
| Yes | 1,670 (48·99) | 1,739 (51·01) |
| Postoperative Dialysis: No | 22,664 (10·46) | 194,070 (89·54) |
| Yes | 436 (41·6) | 613 (58·4) |
| Urinary Tract Infection: No | 21,789 (10·3) | 189,820 (89·7) |
| Yes | 1,311 (21·2) | 4,863 (78·8) |
| Cerebral Vascular Accident/Stroke: No | 22,645 (10·5) | 193,882 (89·5) |
| Yes | 455 (36·2) | 801 (63·8) |
| Cardiac Arrest: No | 22,654 (10·5) | 193,790 (89·5) |
| Yes | 446 (33·3) | 893 (66·7) |
| Myocardial Infarction: No | 22,064 (10·3) | 192,196 (89·7) |
| Yes | 1,036 (29·4) | 2,487 (70·6) |
| Deep Vein Thrombosis/Thrombophlebitis: No | 22,508 (10·5) | 192,806 (89·6) |
| Yes | 592 (24·0) | 1,877 (76·0) |
| Sepsis: No | 21,992 (10·3) | 190,759 (89·7) |
| Yes | 1,108 (22·0) | 3,924 (78·0) |
| Septic Shock: No | 21,124 (9·9) | 191,733 (90·1) |
| Yes | 1,976 (40·1) | 2,950 (59·9) |
| Clostridium difficile infection: No | 22,804 (10·5) | 193,576 (89·5) |
| Yes | 296 (21·1) | 1,107 (78·9) |
| **Postoperative Functional Status** |  |  |
| Independent | 5,081 (4·2) | 115,654 (95·8) |
| Partially Dependent | 10,627 (14·4) | 62,985 (85·6) |
| Totally Dependent | 2,846 (40·2) | 4,243 (59·9) |
| **Services for home discharge** |  |  |
| Discharged to home with services | 4,720 (8·5) | 50,752 (91·5) |
| Discharged to home without services | 4,133 (4·1) | 96,525 (95·9) |
| **Discharge destination** |  |  |
| Home/Permanent Residence | 8,856 (5·7) | 147,301 (94·3) |
| Acute care | 1,011 (28·4) | 2,549 (71·6) |
| Against Medical Advice | 23 (15·1) | 129 (84·9) |
| Other facility | 9,552 (19·1) | 40,362 (80·9) |
| Unknown | 1,779 (47·7) | 1,953 (52·3) |

**Supplemental Table 9.** M**ultivariate logistic regression with respect to the screening probability.** Significant p values shown in bold. p values adjusted using Bonferroni correction.

| **Predictor** | **OR** | **Adjusted 95% CI** | **Adjusted p value** |
| --- | --- | --- | --- |
| Age | 1·002 | [0·999, 1·005] | 0·19 |
| BMI | 1·001 | [0·997, 1·004] | 1·00 |
| Gender |  |  |  |
| Male vs. Female | 0·976 | [0·951, 1·002] | 0·11 |
| Non-binary vs. Female | 0·055 | [0·012, 0·247] | **<0·0001** |
| Race |  |  |  |
| American Indian or Alaska Native vs. White | 0·810 | [0·678, 0·968] | **0·005** |
| Asian vs. White | 0·972 | [0·913, 1·035] | 1·00 |
| Native Hawaiian or Pacific Islander vs. White | 1·020 | [0·782, 1·331] | 1·00 |
| Black or African American vs. White | 0·896 | [0·853, 0·941] | **<0·0001** |
| Other vs. White | 1·076 | [0·978, 1·185] | 0·58 |
| Unknown vs. White | 0·861 | [0·804, 0·923] | **<0·0001** |
| Ethnicity: Hispanic | 0·674 | [0·639, 0·711] | **<0·0001** |
| Obesity | 1·030 | [0·987, 1·075] | 1·00 |
| COPD | 1·022 | [0·976, 1·071] | 1·00 |
| CHF | 1·095 | [1·048, 1·144] | **<0·0001** |
| Bleeding disorder | 0·968 | [0·925, 1·012] | 0·84 |
| Transfusion | 1·079 | [0·976, 1·192] | 0·66 |
| Pre-operative Sepsis | 1·041 | [0·991, 1·094] | 0·38 |
| Hypertension | 1·012 | [0·983, 1·041] | 1·00 |
| Current smoker | 0·901 | [0·853, 0·951] | **<0·0001** |
| Dementia | 1·157 | [1·103, 1·213] | **<0·0001** |
| Fall within last six months | 0·946 | [0·912, 0·982] | **<0·0001** |
| ASA Class |  |  |  |
| 2 – Mild disturbance vs. 1 – No disturbance | 1·388 | [1·075, 1·792] | **0·001** |
| 3 – Severe disturbance vs. 1 – No disturbance | 1·500 | [1·162, 1·936] | **<0·0001** |
| 4 – Life threatening vs. 1 – No disturbance | 1·402 | [1·083, 1·816] | **0·001** |
| 5 – Moribund vs. 1 – No disturbance | 1·141 | [0·812, 1·603] | 1·00 |
| Origin status |  |  |  |
| Acute care hospital vs. Home/Permanent residence | 1·192 | [1·119, 1·270] | **<0·0001** |
| Other facility vs. Home/Permanent residence | 1·160 | [1·055, 1·276] | **<0·0001** |
| Unknown vs. Home/Permanent residence | 0·430 | [0·328, 0·564] | **<0·0001** |
| Pre-operative functional status |  |  |  |
| Partially dependent vs. Independent | 0·947 | [0·901, 0·996] | **0·02** |
| Totally dependent vs. Independent | 0·715 | [0·631, 0·811] | **<0·0001** |
| Urgency: Urgent/Emergency vs. Elective | 1·116 | [1·076, 1·157] | **<0·0001** |
| Surgical specialty |  |  |  |
| Cardiac vs. General surgery | 0·641 | [0·516, 0·795] | **<0·0001** |
| Gynecology vs. General surgery | 0·971 | [0·901, 1·047] | 1·00 |
| Neurosurgery vs. General surgery | 1·241 | [1·176, 1·310] | **<0·0001** |
| Orthopedics vs. General surgery | 1·019 | [0·985, 1·054] | 1·00 |
| Otolaryngology vs. General surgery | 1·076 | [0·969, 1·194] | 1·00 |
| Plastics vs. General surgery | 0·704 | [0·597, 0·830] | **<0·0001** |
| Thoracic vs. General surgery | 1·483 | [1·345, 1·634] | **<0·0001** |
| Urology vs. General surgery | 0·903 | [0·860, 0·948] | **<0·0001** |
| Vascular vs. General surgery | 1·151 | [1·090, 1·215] | **<0·0001** |
| Obstetrics vs. General surgery | 0·884 | [0·491, 1·591] | 1·00 |
| Interventional radiologist vs. General surgery | 0·717 | [0·323, 1·594] | 1·00 |
| Anesthesia type |  |  |  |
| Monitored anesthesia care vs. General | 0·946 | [0·897, 0·997] | **0·02** |
| Epidural vs. General | 1·259 | [0·899, 1·764] | 1·00 |
| Local vs. General | 0·556 | [0·317, 0·976] | **0·03** |
| Regional vs. General | 3·441 | [2·999, 3·948] | **<0·0001** |
| Spinal vs. General | 1·464 | [1·394, 1·538] | **<0·0001** |
| None vs. General | 0·669 | [0·126, 3·569] | 1·00 |
| Other vs. General | 1·665 | [0·992, 2·797] | 0·06 |
| Setting: Outpatient vs. Inpatient | 0·373 | [0·362, 0·383] | **<0·0001** |
| Year |  |  |  |
| 2022 vs. 2021 | 1·0664 | [1·0349, 1·0989] | **<0·0001** |
| 2023 vs. 2021 | 1·3044 | [1·2659, 1·3442] | **<0·0001** |

OR, odds ratio; CI, confidence interval.

**Supplemental Table 10.** **Missingness rates.** Reported as n (%).

| **Characteristic** | **Overall**  **(n = 2,990,064)** | **Delirium**  **(n = 23,100)** | **Non-delirium**  **(n = 194,683)** | **Non-screened**  **(n = 2,772,281)** |
| --- | --- | --- | --- | --- |
| **Demographics** |  |  |  |  |
| Age | 0 (0·0) | 0 (0·0) | 0 (0·0) | 0 (0·0) |
| Age > 90 | 0 (0·0) | 0 (0·0) | 0 (0·0) | 0 (0·0) |
| BMI | 92,281 (3·1) | 2,876 (12·5) | 9,925 (5·1) | 79,480 (2·9) |
| **Gender** | 0 (0·0) | 0 (0·0) | 0 (0·0) | 0 (0·0) |
| **Race** | 0 (0·0) | 0 (0·0) | 0 (0·0) | 0 (0·0) |
| **Ethnicity: Hispanic** | 565,436 (18·9) | 7,816 (33·8) | 50,043 (25·7) | 507,577 (18·3) |
| **ASA Class** | 7,922 (0·3) | 90 (0·4) | 460 (0·2) | 7,372 (0·3) |
| **Preoperative health and comorbidities** |  |  |  |  |
| Obesity | 92,281 (3·1) | 2,876 (12·5) | 9,925 (5·1) | 79,480 (2·9) |
| Diabetes | 0 (0·0) | 0 (0·0) | 0 (0·0) | 0 (0·0) |
| Insulin treated | 0 (0·0) | 0 (0·0) | 0 (0·0) | 0 (0·0) |
| COPD | 0 (0·0) | 0 (0·0) | 0 (0·0) | 0 (0·0) |
| CHF | 0 (0·0) | 0 (0·0) | 0 (0·0) | 0 (0·0) |
| Ascites | 0 (0·0) | 0 (0·0) | 0 (0·0) | 0 (0·0) |
| Dialysis | 0 (0·0) | 0 (0·0) | 0 (0·0) | 0 (0·0) |
| Bleeding disorder | 0 (0·0) | 0 (0·0) | 0 (0·0) | 0 (0·0) |
| Transfusion | 0 (0·0) | 0 (0·0) | 0 (0·0) | 0 (0·0) |
| Pre-operative sepsis | 0 (0·0) | 0 (0·0) | 0 (0·0) | 0 (0·0) |
| Ventilator dependent | 0 (0·0) | 0 (0·0) | 0 (0·0) | 0 (0·0) |
| Hypertension | 0 (0·0) | 0 (0·0) | 0 (0·0) | 0 (0·0) |
| Current smoker | 0 (0·0) | 0 (0·0) | 0 (0·0) | 0 (0·0) |
| Disseminated cancer | 0 (0·0) | 0 (0·0) | 0 (0·0) | 0 (0·0) |
| Corticosteroid use | 0 (0·0) | 0 (0·0) | 0 (0·0) | 0 (0·0) |
| Dementia | 2,517,828 (84·2) | 0 (0·0) | 0 (0·0) | 2,517,828 (90·8) |
| Fall within last 6 months | 2,562,197 (85·7) | 1,670 (7·2) | 13,945 (7·2) | 2,546,582 (91·9) |
| Immunosuppressive Therapy | 0 (0·0) | 0 (0·0) | 0 (0·0) | 0 (0·0) |
| Corticosteroids | 0 (0·0) | 0 (0·0) | 0 (0·0) | 0 (0·0) |
| Anti-rejection/transplant immunosuppressants | 0 (0·0) | 0 (0·0) | 0 (0·0) | 0 (0·0) |
| Synthetic DMARDs/DMDs | 0 (0·0) | 0 (0·0) | 0 (0·0) | 0 (0·0) |
| Biologic DMARDs/DMDs | 0 (0·0) | 0 (0·0) | 0 (0·0) | 0 (0·0) |
| Other | 0 (0·0) | 0 (0·0) | 0 (0·0) | 0 (0·0) |
| **Origin Location** | 0 (0·0) | 0 (0·0) | 0 (0·0) | 0 (0·0) |
| **Preoperative Functional Status** | 32,027 (1·1) | 369 (1·6) | 2,347 (1·2) | 29,311 (1·1) |
| **Home support** | 2,584,026 (86·4) | 4,638 (20·1) | 22,794 (11·7) | 2,556,594 (92·2) |
| **Surgical urgency** | 0 (0·0) | 0 (0·0) | 0 (0·0) | 0 (0·0) |
| **Surgical specialty** | 0 (0·0) | 0 (0·0) | 0 (0·0) | 0 (0·0) |
| **Type of Anesthesia** | 193 (0·0) | 0 (0·0) | 6 (0·0) | 187 (0·0) |
| **Operative setting** | 0 (0·0) | 0 (0·0) | 0 (0·0) | 0 (0·0) |
| **Year** | 0 (0·0) | 0 (0·0) | 0 (0·0) | 0 (0·0) |
| **Time from admission to operation** | 826 (0·0) | 17 (0·1) | 33 (0·0) | 776 (0·0) |
| **Operative time** | 320 (0·0) | 6 (0·0) | 15 (0·0) | 299 (0·0) |
| **Length of Hospital Stay** | 18,674 (0·6) | 1,785 (7·7) | 1,941 (1·0) | 14,948 (0·5) |
| **Mortality within 30 days** | 0 (0·0) | 0 (0·0) | 0 (0·0) | 0 (0·0) |
| **Reoperation** | 1 (0·0) | 0 (0·0) | 0 (0·0) | 1 (0·0) |
| **Readmission** | 0 (0·0) | 0 (0·0) | 0 (0·0) | 0 (0·0) |
| **Unplanned Readmission** | 0 (0·0) | 0 (0·0) | 0 (0·0) | 0 (0·0) |
| **Any Surgical Complication** | 0 (0·0) | 0 (0·0) | 0 (0·0) | 0 (0·0) |
| Superficial Incisional Infection | 0 (0·0) | 0 (0·0) | 0 (0·0) | 0 (0·0) |
| Deep Incisional Infection | 0 (0·0) | 0 (0·0) | 0 (0·0) | 0 (0·0) |
| Organ Space Infection | 0 (0·0) | 0 (0·0) | 0 (0·0) | 0 (0·0) |
| Dehiscence | 0 (0·0) | 0 (0·0) | 0 (0·0) | 0 (0·0) |
| Bleeding/Transfusion | 0 (0·0) | 0 (0·0) | 0 (0·0) | 0 (0·0) |
| Postop total transfusion amount | 2,872,646 (96·1) | 18,383 (79·6) | 177,748 (91·3) | 2,676,515 (96·6) |
| **Any Medical Complication** | 2,638,953 (88·3) | 0 (0·00) | 0 (0·00) | 2,638,953 (95·2) |
| Pneumonia | 0 (0·0) | 0 (0·0) | 0 (0·0) | 0 (0·0) |
| Pulmonary embolism | 0 (0·0) | 0 (0·0) | 0 (0·0) | 0 (0·0) |
| Reintubation | 0 (0·0) | 0 (0·0) | 0 (0·0) | 0 (0·0) |
| Ventilator Dependence >48h | 0 (0·0) | 0 (0·0) | 0 (0·0) | 0 (0·0) |
| Postoperative Dialysis | 0 (0·0) | 0 (0·0) | 0 (0·0) | 0 (0·0) |
| Urinary Tract Infection | 0 (0·0) | 0 (0·0) | 0 (0·0) | 0 (0·0) |
| Cerebral Vascular Accident/Stroke | 0 (0·0) | 0 (0·0) | 0 (0·0) | 0 (0·0) |
| Cardiac Arrest | 0 (0·0) | 0 (0·0) | 0 (0·0) | 0 (0·0) |
| Myocardial Infarction | 0 (0·0) | 0 (0·0) | 0 (0·0) | 0 (0·0) |
| Deep Vein Thrombosis/Thrombophlebitis | 0 (0·0) | 0 (0·0) | 0 (0·0) | 0 (0·0) |
| Sepsis | 0 (0·0) | 0 (0·0) | 0 (0·0) | 0 (0·0) |
| Septic Shock | 0 (0·0) | 0 (0·0) | 0 (0·0) | 0 (0·0) |
| Clostridium difficile infection | 0 (0·0) | 0 (0·0) | 0 (0·0) | 0 (0·0) |
| **Postoperative Functional Status** | 2,531,036 (84·7) | 4,546 (19·7) | 11,801 (6·1) | 2,514,689 (90·7) |
| **Services for home discharge** | 2,622,794 (87·7) | 14,247 (61·7) | 47,406 (24·4) | 2,561,141 (92·4) |
| **Discharge destination** | 17,536 (0·6) | 1,879 (8·1) | 2,389 (1·2) | 13,268 (0·5) |
